# The mRNA and protein levels of the glycolytic enzymes lactate dehydrogenase A (LDHA) and phosphofructokinase platelet (PFKP) are good predictors of survival time, recurrence, and risk of death in cervical cancer patients

**DOI:** 10.1002/cam4.6123

**Published:** 2023-06-16

**Authors:** Verónica Bolaños‐Suárez, Ana Alfaro, Ana María Espinosa, Ingrid Medina‐Martínez, Eligia Juárez, Nicolás Villegas‐Sepúlveda, Marco Gudiño‐Zayas, América Gutiérrez‐Castro, Edgar Román‐Bassaure, María Eugenia Salinas‐Nieves, Sergio Bruno‐Muñoz, Carlos Aranda, Oscar Flores‐Herrera, Jaime Berumen

**Affiliations:** ^1^ Unidad de Investigación en Medicina Experimental, Facultad de Medicina Universidad Nacional Autónoma de México Mexico City Mexico; ^2^ Posgrado en Ciencias Biológicas, Unidad de Posgrado, Edificio D, 1° Piso, Circuito de Posgrados Universidad Nacional Autónoma de México Mexico City Mexico; ^3^ Anatomía Patológica Hospital General de México, Dr. Eduardo Liceaga Mexico City Mexico; ^4^ Farmacología Clínica Hospital General de México, Dr. Eduardo Liceaga Mexico City Mexico; ^5^ Departamento de Bioquímica y Biología Molecular, Facultad de Medicina Universidad Nacional Autónoma de México Mexico City Mexico; ^6^ Unidad de Medicina Genómica Hospital General de México, Dr. Eduardo Liceaga Mexico City Mexico; ^7^ Departamento de Biomedicina Molecular Centro de Investigación y Estudios Avanzados del Instituto Politécnico Nacional Mexico City Mexico; ^8^ Servicio de Oncología Hospital General de México, Dr. Eduardo Liceaga Mexico City Mexico; ^9^ Servicio de Ginecología, Clínica de Colposcopia Hospital General de México Dr. Eduardo Liceaga Mexico City Mexico; ^10^ Departamento de Bioquímica, Facultad de Medicina Universidad Nacional Autónoma de México Mexico City Mexico

**Keywords:** cervical cancer, glycolysis, lactate dehydrogenase A (LDHA), phosphofructokinase platelet (PFKP), risk of death, survival

## Abstract

**Introduction:**

Patients with cervical cancer (CC) may experience local recurrence very often after treatment; when only clinical parameters are used, most cases are diagnosed in late stages, which decreases the chance of recovery. Molecular markers can improve the prediction of clinical outcome. Glycolysis is altered in 70% of CCs, so molecular markers of this pathway associated with the aggressiveness of CC can be identified.

**Methods:**

The expression of 14 glycolytic genes was analyzed in 97 CC and 29 healthy cervical tissue (HCT) with microarray; only LDHA and PFKP were validated at the mRNA and protein levels in 36 of those CC samples and in 109 new CC samples, and 31 HCT samples by qRT–PCR, Western blotting, or immunohistochemistry. A replica analysis was performed on 295 CC from The Cancer Genome Atlas (TCGA) database.

**Results:**

The protein expression of LDHA and PFKP was associated with poor overall survival [OS: LDHA HR = 4.0 (95% CI = 1.4–11.1); *p* = 8.0 × 10^−3^; PFKP HR = 3.3 (95% CI = 1.1–10.5); *p* = 4.0 × 10^−2^] and disease‐free survival [DFS: LDHA HR = 4.5 (95% CI = 1.9–10.8); *p* = 1.0 × 10^−3^ *;* PFKP HR = 3.2 (95% CI = 1.2–8.2); *p* = 1.8 × 10^−2^] independent of FIGO clinical stage, and the results for mRNA expression were similar. The risk of death was greater in patients with overexpression of both biomarkers than in patients with advanced FIGO stage [HR = 8.1 (95% CI = 2.6–26.1; *p* = 4.3 × 10^−4^) versus HR = 7 (95% CI 1.6–31.1, *p* = 1.0 × 10^−2^)] and increased exponentially as the expression of LDHA and PFKP increased.

**Conclusions:**

LDHA and PFKP overexpression at the mRNA and protein levels was associated with poor OS and DFS and increased risk of death in CC patients regardless of FIGO stage. The measurement of these two markers could be very useful for evaluating clinical evolution and the risk of death from CC and could facilitate better treatment decision making.

## Graphical abstract

Glycolysis is a metabolic pathway in which molecular markers associated with the survival and risk of death of patients with cervical cancer (CC) can be identified. Lactate dehydrogenase A (LDHA) and phosphofructokinase platelet (PFKP) were associated with poor overall survival and disease‐free survival, the risk of death was greater when both biomarkers were overexpressed than when using only FIGO stage. LDHA and PFKP could be good biomarkers and potential therapeutic targets in CC.

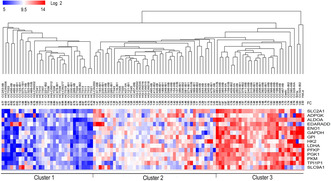

## 1 INTRODUCTION

Cervical cancer (CC) is the fourth most common cancer in women worldwide.^1^ Persistent infection with human papillomavirus (HPV), particularly high‐risk oncogenic viruses, is the main etiological factor for the development of CC. Despite early detection programs and vaccinations against most oncogenic HPVs,^2^ it is estimated that 569,000 new cases and 311,365 related deaths occur each year worldwide^1^; thus, CC continues to be a major health problem, mainly in developing countries where most cases occur. CC results from the progression of cervical intraepithelial neoplasms (CINs), which are histologically classified into low grade (LG‐CIN) and high grade (HG‐CIN). CC originates mainly from HG‐CINs.^3^

Treatment of CC includes surgery, radiotherapy, and chemotherapy and depends on the clinical stage of the disease.^4^ However, it is estimated that a significant percentage of patients have pelvic recurrence (10–23% in stage IB‐IIB and 42–74% in stage III‐IVA) or metastases (16–26% in stage IB‐IIB and 39–75% in stage III‐IVA) after treatment, which often worsens the prognosis.^5^, ^6^ In addition, only 32% of cases of recurrent disease are identified early (before 6 months) during medical follow‐up, which decreases the chance of recovery and the survival time.^7^ Although the clinical International Federation of Gynecology and Obstetrics (FIGO) stage, the clinical characteristics of the tumor, metastasis to lymph nodes, and parametrial invasion are predictors of recurrence and survival time, there are no molecular markers approved for clinical use that predict the clinical evolution of CC in patients. Molecular markers alone or in conjunction with clinical data can improve the prediction of the clinical outcome and facilitate better therapeutic decision‐making, as has been demonstrated in colorectal^8^ and breast^9^ cancers.

Since glycolysis is increased in 70% of human cancers, and lactate production occurs even in the presence of oxygen (the Warburg effect),^10^, ^11^ glycolysis pathways can be explored to identify new prognostic biomarkers in CC. In fact, the usefulness of several genes, proteins, or other variables related to glycolysis has been investigated for the evaluation of survival and disease aggressiveness in CC; these factors include glucose transporter 1 (GLUT1),^12^ hexokinase 2 (HK2),^13^ phosphofructokinase isoform M (PFKM) (as part of a genetic profile),^14^ and total lesion glycolysis (TLG), a parameter measurable through positron emission tomography (PET).^15^ However, the predictive efficacy of these biomarkers either has not been reported or has been reported to be intermediate. While PET could be considered the most efficient method for biomarker detection, it is very expensive. On the other hand, it would be desirable to identify molecular therapeutic targets in CC as the antitumor strategy with specific target drugs is showing great benefit in many tumor types with a marked decrease in side effects.^16^

In a previous study, our group found that a glycolytic gene profile in CC was associated with a decrease in survival.^17^ In this paper, we investigated which genes of the glycolytic expression profile are most highly associated with survival and tumor aggression in CC. From the 14 genes explored in the discovery set (*n* = 97 CC samples), only two genes were associated with OS and DFS independent of FIGO clinical stage: lactate dehydrogenase A (*LDHA*) and phosphofructokinase platelet (*PFKP*). They were validated at the mRNA and protein levels in 36 CC samples of the discovery set and in 109 new CC samples by qRT–PCR, Western blotting, or immunohistochemistry. In addition, a replica analysis was performed for 295 CC samples from The Cancer Genome Atlas (TCGA) database. These two markers could allow us to evaluate the clinical evolution and prognosis of patients with CC regardless of FIGO stage.

## 2 MATERIALS AND METHODS

### 2.1 Patient selection and clinical characteristics

The study included 206 patients with CC (Figure S1, Table S1), 10 patients with high‐grade cervical intraepithelial neoplasms (HG‐CIN), and 60 women providing healthy cervical tissue (HCT) evaluated in the Departments of Oncology and Gynecology and Obstetrics of the HGM. Patients with CC were selected from a previous study that included 462 patients recruited from November 2003 to April 2005 and from January 2006 to July 2007.^19^ The inclusion criteria were as follows: patients diagnosed with invasive CC and no previous treatments. Only patients whose high‐quality RNA and tumor biopsy samples had more than 70% tumor cells were included in the present study. The exclusion criteria were insufficient quality of the biological sample. All patients received complete clinical evaluation and were treated with surgery, radiation, chemotherapy, or a combination of these according to American Cancer Society guidelines. Tumor staging was performed in accordance with the latest FIGO protocol for gynecological cancer.^20^ The average age of the patients with CC was 51 ± 14 years, and that of the patients with HG‐CIN was 39.3 ± 10.5 years. After treatment, patients were followed up and evaluated periodically at the HGM. HCT samples were obtained from patients who underwent hysterectomy for myomatosis with a normal cervix according to cytology and colposcopy as described previously.^19^ The average age of women providing HCT samples was 46.2 ± 3.1.

### 2.2 RNA isolation

Total RNA from the samples was extracted with TRIzol™ reagent (Invitrogen, Carlsbad CA, USA) according to the manufacturer's instructions. RNA integrity was verified by agarose gel electrophoresis according to the ratio of 28S to 18S ribosomal RNA.

### 2.3 DNA isolation

The DNA was extracted using the PureLink Genomic DNA Kit (Invitrogen, Carlsbad, CA, USA).

### 2.4 HPV detection and typing

HPV detection was performed by PCR using universal primers located in the HPV L1 gene (*MY09*/*MY11*, *GP5+*/*6+*, and *L1C1*), as described previously.^19^ The *HBB* gene was used as an internal control to assess the quality of the DNA. The HPV types were identified by sequencing the amplified bands using the fluorescent cycle‐sequencing method (BigDye Terminator Ready Reaction Kit; Applied Biosystems, Carlsbad, CA, USA). Sequence analysis was performed using an ABI PRISM 3130xl Genetic Analyzer system (Applied Biosystems). Each band sequenced was analyzed with the FASTA sequence similarity. The average identity percentage of HPV types detected was 98.7% (91–100%) when compared to the reference sequences.

### 2.5 Glycolytic gene expression and data analysis

Glycolytic gene expression was examined by microarray in 97 CC, 10 HG‐CIN, and 29 HCT samples [76 CC, 10 HG‐CIN and 17 HCT samples by Human Gene 1.0 ST (HG‐1.0 ST) and 42 CC and 12 HCT samples by Human Gene Focus (HG‐Focus) (Affymetrix, Santa Clara, CA), with 21 CC samples in common]. Gene expression data were deposited in the Gene Expression Omnibus (GEO) database with accession numbers GSE52904^17^ and GSE39001.^19^

HG‐1.0 ST was standardized with the robust multiarray average algorithm in the Affymetrix expression console, and HG‐Focus was standardized with the robust multichip average algorithm of FlexArray software.^17^, ^19^ The identification of differentially expressed glycolytic genes between CC and HCT was performed with the SAM algorithm (SAM version 3.0, http://statweb.stanford.edu/~tibs/SAM/) using a fold change (FC) cutoff value of ≥1.5, a general false discovery rate of 0%, and a local false discovery rate of ≤10%. The normalized intensity values were log_2_‐transformed for analysis. We identified 14 glycolytic genes that met the selection criteria: *SLC2A1*, *ADPGK*, *HK2*, *GPI*, *PFKP*, *ALDOA*, *TPI1P1*, *GAPDH*, *PGK1*, *ENO1*, *PKM*, *LDHA*, *SLC9A1*, and *EDARADD*. We performed an unsupervised hierarchical grouping analysis using dChip software (version 1.6, http://www.hsph.harvard.edu/cli/complab/dchip/) with the parameters of Euclidean metric distance, linkage average method, genes ordered by the time peak, and rows standardized by the mean.^17^ In the hierarchical analysis, the samples were segregated into clusters based on the main branches of the dendrogram.

A glycolysis FC score model was constructed. For the 14 glycolytic genes studied, the FC was calculated by dividing the normalized intensity values of each sample by the average normalized intensity values of the control sample group (HCT). Then, for each sample, the median FC of the 14 glycolytic genes was calculated and considered the glycolysis FC score.

### 2.6 Quantitative PCR (qRT–PCR)

cDNA was synthesized using the High‐Capacity cDNA Transcription Kit (Applied Biosystems) using 2 μg of RNA according to the manufacturer's protocol. Gene expression of *LDHA*, *PFKP*, and an internal control (*RPS13*) was measured in 58 CC (14 from the discovery sample and 44 new CC) and 19 HCT by qRT–PCR, and TaqMan gene expression assays were used (*LDHA*, Hs00855332_g1; *PFKP*, Hs00242993_m1; *RPS13*, HS 01011487_g1; Applied Biosystem Inc.). The experiments were run in triplicate in a final volume of 20 μL, including 200 ng of cDNA template using TaqMan® Universal PCR Master Mix (4304437, Applied Biosystems), according to the manufacturer's instructions. The expression was normalized with respect to the internal control (*RPS13*) and the HCT group by the double delta method (2 ^−ΔΔCT^) as previously reported.^21^ The FC in expression was calculated by dividing the median normalized intensity of each tumor sample by the median normalized intensity of all HCT samples.

### 2.7 Western blotting (WB)

LDHA and PFKP protein expression was determined using WB in 69 CC samples (22 from the discovery sample and 47 new CC). Twenty‐five nanograms of protein was resolved by 8% SDS–PAGE, electrotransferred onto a nitrocellulose membrane and incubated with a mouse monoclonal antibody anti‐human LDHA (H‐10: sc‐133123; 1:1,000) or PFKP (F‐7: sc‐514824; 1:200) and goat β‐actin antibody (I‐19: sc‐1616) (Santa Cruz Biotechnology, Inc.) overnight at 4°C. The membranes were then incubated with horseradish peroxidase (HRP)‐conjugated secondary antibodies (anti‐mouse IgG + IgM (H + L) antibodies; 1:10,00; Jackson ImmunoResearch Laboratories, Inc.) and anti‐goat IgG‐HRP (sc‐2354; 1:1,000; Santa Cruz Biotechnology, Inc.) for 1 h at room temperature. Prestained broad range SDS–PAGE standards (BIO‐RAD, CA) were used for molecular weight estimation on gels. β‐Actin was used as an internal control. Loading buffer without sample was used as a negative control. The immunoreactive proteins were developed using the SuperSignal™ Chemiluminescent HRP Kit (Thermo Fisher Scientific). Densitometric analysis was performed with ImageJ software (NIH, Bethesda, MD). The measurement of density profiles and background correction were performed with the default settings of the software. The size of the analyzed areas was the same for all the bands.^22^ The optical density was calculated as OD = log_10_(255/pixel value).

### 2.8 Immunohistochemistry (IHC)

The protein expression of LDHA and PFKP was determined in 12 HCT, 18 CC, and 6 metastatic samples by IHC. Human paraffin‐embedded tissue samples were collected at the Pathology Department of HGM from patients evaluated from January 2008 to March 2013. The inclusion criteria were as follows: CC at any FIGO stage, diagnostic biopsy prior to treatment, complete clinical data, and follow‐up data for at least 24 months after treatment. All patients received complete clinical evaluation according to the ACS guidelines. Clinicopathological information was collected from medical records. Tissue microarrays (TMAs) were built as previously described.^19^ Serial sections (4 μm thick) of the TMA were cut, and the tenth slide was stained with Hematoxylin and Eosin to confirm the histopathological diagnosis by two pathologists. IHC was performed with the Ultra Streptavidin (USA) HRP Detection kit (Multi‐Species) (BioLegend, CA) according to the manufacturer's instructions. The following mouse monoclonal antibodies were used: LDH (H‐10) sc‐133123 (1:200) and PFKP (F‐7) sc‐514824 (1:100) from Santa Cruz Biotechnology (Santa Cruz, CA). Antigen–antibody complexes were detected using the avidin‐biotin peroxidase method, with 3,3′‐diaminobenzidine‐tetrahydrochloride (DAB) as a chromogenic substrate (DAB Chromogen Concentrate, BioLegend, CA), and the sections were counterstained with hematoxylin. Assays were performed in triplicate.

### 2.9 Quantitative image analysis

Each tissue of the TMA was photographed in triplicate at a magnification of 400X using a Nikon Microphot‐FXA. The digital images were analyzed with ImageJ software. The immunoreactivity of LDHA and PFKP was analyzed with Ruifrok and Johnston's color deconvolution method as previously described.^23^ In each image, the intensity (pixels) and area stained with chromogen were determined. The intensity of the DAB signal was transformed to optical density values: OD = −log (255* maximum level pixels)/average pixels. The integrated optical density (DOI) was calculated as the OD × staining area.

### 2.10 Survival analysis

After the treatment was completed, each patient was clinically evaluated every 3 or 6 months by an experienced oncologist. Clinical follow‐up data were obtained from the patient's medical record. Additionally, a social worker called the patients and visited their homes every 6 months during the study. Survival analysis was performed on all patients who received the full treatment. The mean follow‐up time was 60 months after the initial diagnosis. The patients designated “censored” are patients who were lost to follow‐up or who died from causes other than CC. Patients were considered lost to follow‐up when they did not attend medical appointments for disease control, were not at home during visits or did not answer phone calls. In this cohort, survival status was recorded based on the last follow‐up, and death caused by a primary CC tumor was recorded when confirmed by the medical record and the death certificate.

### 2.11 Analysis of the TCGA database

Raw gene expression data (RNAseq) of 12 genes involved in glycolysis (*SLC2A1*, *ADPGK*, *HK2*, *GPI*, *PFKP*, *ALDOA*, *GAPDH*, *PGK1*, *ENO1*, *LDHA*, *SLC9A1*, and *EDARADD*), which were measured in 295 CC and 3 HCT samples, were obtained from the TCGA database (https://cancergenome.nih.gov/) using the UCSC Xena web tool (https://xena.ucsc.edu).^24^ The normalized intensity values were log_2_‐transformed for the analysis. These data were used to perform an unsupervised hierarchical grouping analysis using dChip software according to the parameters described above. Additionally, we explored the relationship between the gene expression of *LDHA* and *PFKP* genes with OS and with the risk of death using the Kaplan–Meier method and Cox proportional hazards regression models, respectively. The multivariate Cox models also included the FIGO clinical stage to investigate whether the effect of gene expression on the risk of death is independent of the clinical stage.

### 2.12 Gene ontology classification analysis

The Transcriptome Analysis Console (TAC) v4.0.3 (Thermo Fisher Scientific, Inc., Waltham, MA, USA) was used to identify genes differentially expressed between CC samples and HCT samples. The CC group was divided into two groups according to the median glycolysis FC score of 1.42. The DAVID functional annotation tool (http://david.abcc.ncifcrf.gov/) was utilized to classify the deregulated genes via functional annotation clustering considering the gene ontology biological processes. The classification stringency level was set to medium.

### 2.13 Statistical analysis

Data analysis were performed using SPSS software ver. 20. Receiver operating characteristic (ROC) curve analysis was performed, and the Youden index was used^25^ to select the best cutoff points of gene expression for overall survival (OS) or disease‐free survival (DFS) analysis. Genes and proteins with expression values equal to or above the cutoff were considered upregulated, and those with values below the cutoff were considered downregulated.

The comparisons of OS and DFS between patients in the high and low tumor gene and protein expression groups were performed by the Kaplan–Meier method, and the significance of differences was calculated by the log‐rank test. FIGO staging and glycolysis gene expression were included in univariate and multivariable Cox proportional hazards regression models. All tests were two‐sided, and *p* values less than 0.05 were considered to indicate statistical significance.

## 3 RESULTS

### 3.1 Analysis of glycolytic gene expression in CC

**FIGURE 1:**
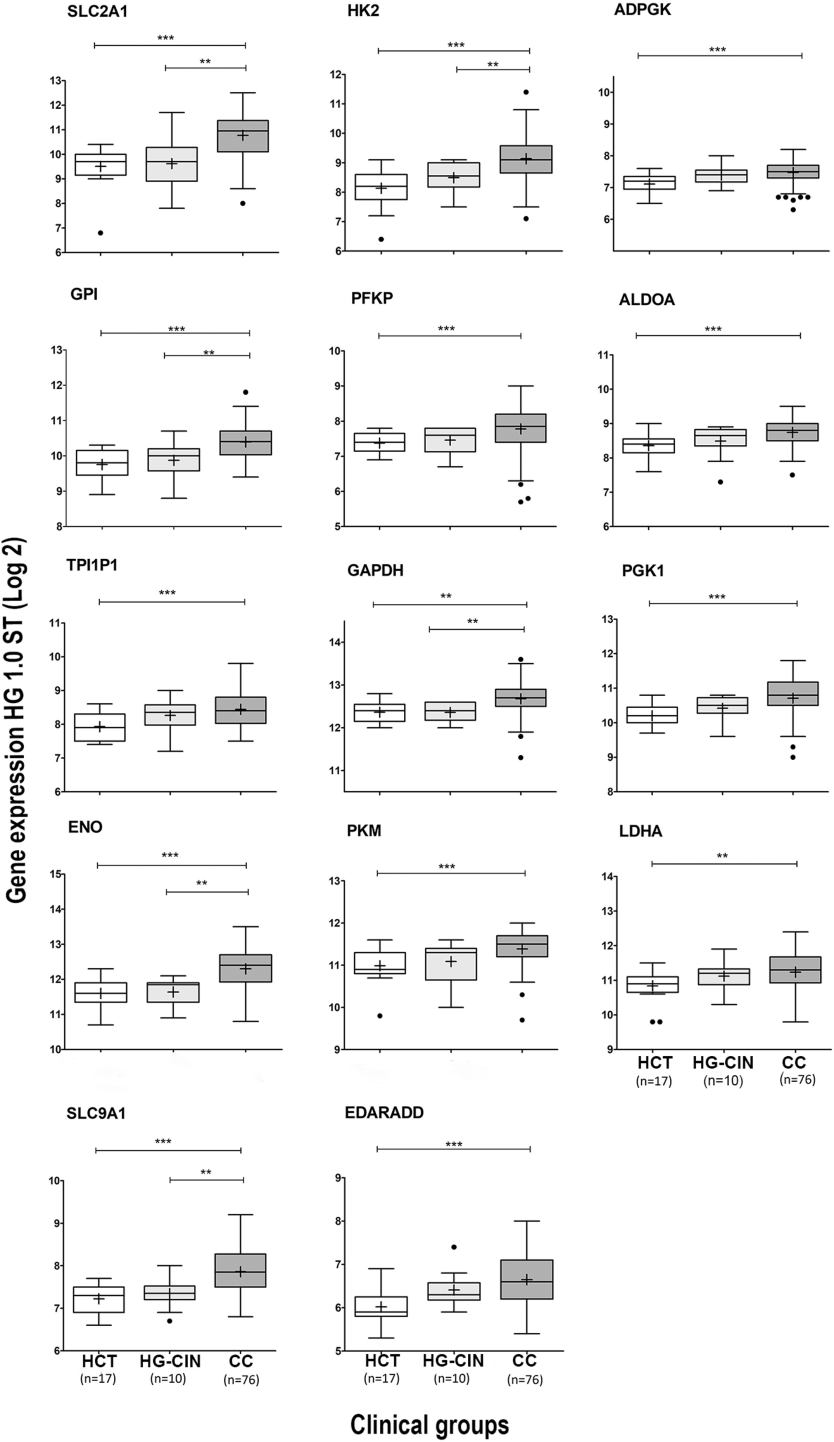
Box plots of the expression of 14 genes of the glycolytic pathway obtained with the HG‐1.0 ST microarray. The analysis was performed on 17 HCT, 10 HG‐CIN, and 76 CC samples. The graphs show the value of the normalized fluorescence intensity (log 2) for each gene. The upper and lower limits of the boxes represent the 75th and 25th percentiles, respectively. The mean is shown as the center black line inside the boxes, and the median is shown as “+”. The whiskers represent the maximum and minimum values that lie within 1.5 times the interquartile range from the ends of the frame. Values outside this range are displayed as black dots. The *Mann–Whitney U test* was used to determine the significant differences between the groups, **p < 0.05, ***p < 0.005. CC, cervical cancer; HCT, healthy cervical tissue; HG‐CIN, high‐grade cervical intraepithelial neoplasm.

In the HG‐1.0 ST microarray analysis, 14 genes involved in glycolysis had significantly higher expression levels in the CC tumors than in control samples (Figure 1). We were able to confirm the difference in expression for 9 of these genes (*SLC2A1*, *HK2*, *PFKP*, *ALDOA*, *GAPDH*, *PGK1*, *ENO1*, *PKM*, and *LDHA*) in a second microarray with 42 invasive CCs and 12 HCTs (HG‐Focus; see Figure S2); the dataset for this microarray only included information on 9 of the 14 formerly explored genes.

**FIGURE 2:**
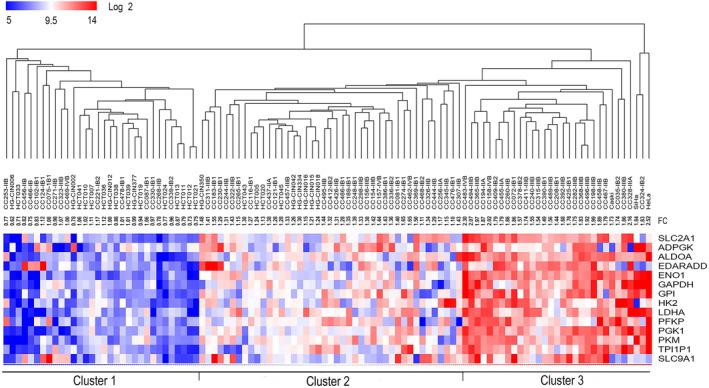
Unsupervised hierarchical cluster analysis of 14 glycolytic genes in CC. The segregation of HCT (*n* = 17), HG‐CIN (*n* = 10), and CC (*n* = 76) samples is shown according to the expression profile of 14 glycolytic genes. Three clusters were distinguished: the first cluster had a downregulated glycolysis profile, the second had an intermediate expression glycolysis profile, and the third cluster had an upregulated glycolysis profile. The intensity of gene expression was coded as follows: red for upregulation, blue for downregulation, and white for no change in expression. Each column represents a sample, and each line represents a glycolytic gene. The number at the end of the CC sample name indicates the FIGO stages of the patient. The analysis was performed with the expression values expressed in base 2 logarithmic. The glycolysis FC score was indicated for each sample (FC). CC, cervical cancer; HCT, healthy cervical tissue; HG‐CIN, high‐grade cervical intraepithelial neoplasm.

The samples were distributed into three clusters according to expression profile in the hierarchical analysis: upregulation (Cluster 3), intermediate regulation (Cluster 2), and downregulation (Cluster 1). Cluster 3 showed upregulation of most of the genes of glycolysis and was composed only of CC samples (*n* = 28) and the three CC‐derived cell lines (HeLa, SiHa, and CaSki), which could be considered to exhibit the highest degree of neoplastic aggressiveness. In contrast, in Cluster 1, in which glycolysis genes were not overexpressed, almost all controls (*n* = 13, 76.5%), 4 of the 10 HG‐CINs, and a group of 15 CCs (19.7%) were included. The rest of the controls, HG‐CINs and CC were distributed in Cluster 2. However, the CCs in Cluster 2 were not arranged as expected according to the glycolysis FC score. Some tumors with higher scores were closer to Cluster 1, whereas some with low scores were closer to Cluster 3 (Figure 2, Table S2).

In the hierarchical analysis of samples on the HG‐Focus microarray, the distribution of the CCs and controls was very similar to the distribution obtained with the HG‐1.0 ST data (Figure S3, Table S2).

The distribution of the CCs according to the clinical stage (≤IIA versus ≥ IIB) was not different among the clusters with different glycolysis gene expression profiles, either in the study carried out with the HG‐1.0 ST or with the HG‐Focus microarray (Table S2). We found no differences between CCs positive for HPV16 and other HPVs in the hierarchical analysis (*p* = 1.6 × 10^−1^, chi‐square test) (Table S2).

### 3.2 Effect of the expression of 14 glycolysis genes on survival

**FIGURE 3:**
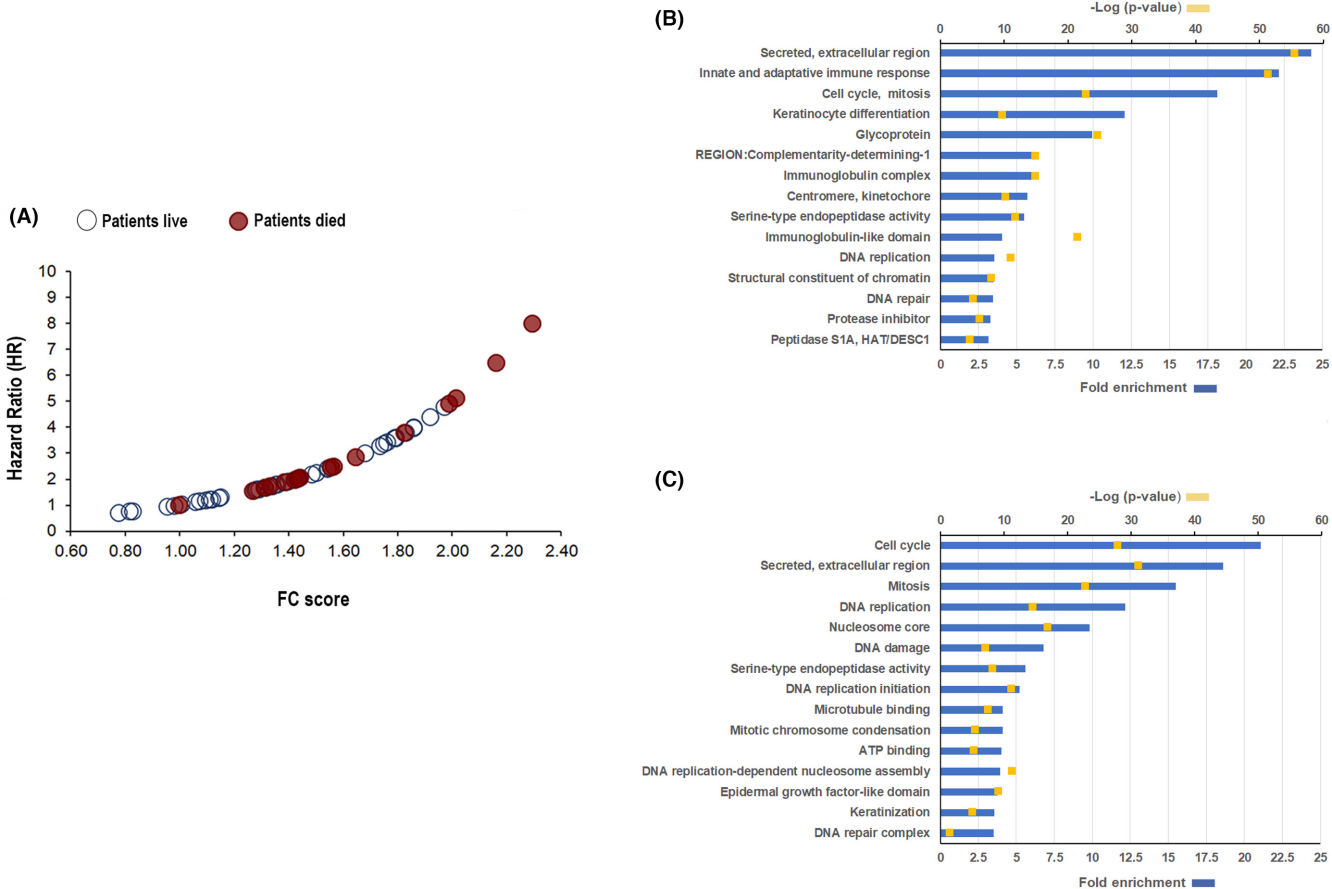
Hazard ratio (HR) analysis and identification of biological processes enriched in CC according to the glycolysis fold change (FC) score. Panel (A) shows the hazard ratio (HR) analysis in relation to the glycolysis FC score obtained with the data of the HG‐1.0 ST microarray. The risk of death from CC increases exponentially as glycolytic gene expression increases. Red circles represent dead patients, while light circles represent surviving patients. Panels (B and C) show the top 15 canonical pathways enriched in CC with glycolysis FC scores <1.42 (*n* = 30) and ≥1.42 (*n* = 31), respectively. Fold enrichment (blue bars) and *p values* (yellow squares) were obtained with the DAVID functional annotation tool (see Materials and Methods).

The 5‐year survival progressively decreased as glycolysis‐related gene expression increased from Cluster 1 to 3. The survival rate decreased 8% in Cluster 1, 26% in Cluster 2, and 44% in Cluster 3 patients (*p* = 0.075, log‐rank test; Figure S4A). This progressive association between the glycolysis profile and survival was better illustrated using the glycolysis FC score, which is a continuous variable, in the Cox model: the risk of death (HR) increased 4.96 times for each increase of one FC score unit [HR = 4.96 (95% CI 1.1–22.2; *p* = 3.6 × 10^−2^, Cox test)]. The HR for each patient was calculated according to their FC score, and the risk of death (HR) increased exponentially with increases in the FC score (see Figure 3A). At an FC = 1.42 (median glycolysis FC score), the risk of death was almost twofold higher (HR = 1.96) than when the FC score = 1; with the maximum FC score observed (2.3), the HR was approximately 8. This suggests that the overexpression of glycolysis‐related genes could be a poor prognostic factor for CC.

In fact, in the analysis of global gene expression, the biological processes linked to cancer were more enriched in tumors with FC scores ≥1.42 (Figure 3C) than in those with FC scores below 1.42; in the latter group, the enrichment of processes linked to the cellular and humoral immune response was observed (Figure 3B). These data could explain the difference in clinical behavior between these two groups of tumors.

To investigate which genes, contribute most significantly to that profile, the survival was analyzed separately for each gene according to the best cutoff calculated with the ROC analysis. Of the 14 genes identified with the HG‐1.0 ST microarray, the overexpression of only 8 (*GAPDH*, *PGK1*, *TPI1P1*, *LDHA*, *ALDOA*, *PFKP*, *ENO1*, and *GPI*) was significantly associated with poor OS of the patients, with *p* values ranging from *p* = 1.0 × 10^−4^ to *p* = 1.3 × 10^−2^ in the log‐rank test (Figure S4B–I). In addition, five of them (*GPI*, *PFKP*, *TPI1P1*, *GAPDH*, and *LDHA*) were associated with a significant reduction in % DFS (Figure S4J–O). Of the nine genes explored via HG‐Focus microarray analysis, the gene expression levels of only three of them (*ALDO*, *PGK1*, and *LDHA*) were significantly associated with the reduction in OS rate (*p* = 3.3 × 10^−2^, *p* = 9.0 × 10^−3^, and *p* = 5.0 × 10^−2^, respectively, log‐rank test), and only one (*LDHA; p* = 3.0 × 10^−2^, log‐rank test) was associated with DFS (see Figure S5).

To determine whether the effect of overexpression of these genes on survival was independent of clinical stage, both variables were analyzed in a multivariate Cox proportional hazards model. Due to the small number of patients (*n* = 61), they were grouped into two clinical groups, Group 1 (≤ stage IIA, *n* = 29) and Group 2 (≥ stage IIB, *n* = 32). Univariate analysis showed that the risk of death (HR) of patients in Group 2 was 3.4 [95% confidence interval (CI) = 1.1–10.4; *p* = 3.6 × 10^−2^, Cox test; Table 1] times higher than that of patients in Group 1. As expected, the overexpression of seven of the eight genes conferred an increased risk of death, ranging from an HR of 2.8 (95% CI 1.0–7.6; *p* = 3.7 × 10^−2^) for the *PGK1* gene to an HR of 9.2 (95% CI = 1.2–69.5; *p* = 3.7 × 10^−2^) for the *ENO1* gene (see Table 1). However, when explored in conjunction with FIGO stage in the multivariate analysis, only the *LDHA* gene with an HR of 3.0 (95% CI = 1.1–8.2; *p* = 2.9 × 10^−2^), the *PFKP* gene with an HR of 3.4 (95% CI = 1.1–10.5; *p* = 3.5 × 10^−2^), and the pseudogene *TPI1P1* with an HR of 2.6 (95% CI = 1.0–7.9; *p* = 4.0 × 10^−2^) conferred an increased risk of death independent of FIGO clinical stage (Table 1). When DFS was analyzed, only *LDHA*, with an HR of 2.7 (95% CI = 1.1–6.2; *p* = 2.9 × 10^−2^), had a FIGO stage‐independent effect (Table S3). *LDHA* experimental findings were also confirmed with the HG‐Focus data (see Tables S4 and S5).

**TABLE 1:** Univariate and multivariate analyses of factoring affecting the OS of patients with CC based on Cox proportional hazards models including the expression of glycolytic genes explored with the HG‐1.0 ST microarray and FIGO clinical stage.

Covariates	*n*	Univariate analysis^g^			Multivariate analysis^h^		
		HR^d^	95% CI	*p* ^e^	HR^b^	95% CI	*p* ^e^
FIGO							
≤IIA	29	1.0			1.0		
≥IIB	32	3.4	1.1–10.4	3.6 × 10^−2^	3.3^d^	1.1–9.6^d^	3.5 × 10^−2^ ^f^
Glycolytic gene expression profile							
FC <1.98^a^	57	1.0			1.0		
FC≥1.98	4	8.6	2.7–28.0	3.2 × 10^−4^	5.8	1.7–20.2	5.0 × 10^−3^
Glycolysis FC score^b^	61	5.0	1.1–22.3	3.6 × 10^−2^	3.2	1.0–14.5	1.2 × 10^−2^
*LDHA*							
Low^c^	40	1.0			1.0		
High	21	3.1	1.1–8.3	2.7 × 10^−2^	3.0	1.1–8.19	2.9 × 10^−2^
*PFKP*							
Low	32	1.0			1.0		
High	29	3.5	1.1–10.9	3.0 × 10^−2^	3.4	1.1–10.5	3.5 × 10^−2^
*TPI1P1*							
Low	52	1.0			1.0		
High	9	3.7	1.3–10.9	1.5 × 10^−3^	2.6	0.8–7.87	4.0 × 10^−2^
*GAPDH*							
Low	54	1.0			1.0		
High	7	5.6	1.9–16.3	2.0 × 10^−3^	4.0	1.3–12.3	5.2 × 10^−2^
*GPI*							
Low	19	1.0			1.0		
High	42	8.5	1.1–64.1	3.9 × 10^−2^	7.9	1.0–60.0	5.6 × 10^−2^
*ENO*							
Low	21	1.0			1.0		
High	40	9.2	1.2–69.5	3.2 × 10^−2^	7.3	0.9–56.4	5.6 × 10^−2^
*PGK1*							
Low	44	1.0			1.0		
High	17	2.8	1.0–7.6	3.7 × 10^−2^	2.3	0.9–6.2	1.1 × 10^−1^
*ALDOA*							
Low	15	1.0			1.0		
High	46	5.8	0.7–44.3	8.7 × 10^−2^	4.9	0.6–37.6	1.2 × 10^−1^

Abbreviations: CI, confidence interval; FIGO stage, International Federation of Gynecology and Obstetrics stage; HR, hazard ratio.

^a^ Optimal cutoff values were selected according to the ROC curve of the glycolysis FC score.

^b^ The analysis was performed considering the glycolysis FC score as continuous variable.

^c^ Optimal cutoff values were selected according to the ROC analysis in relation to fold changes in genes expression obtained with the Human Gene 1.0 ST microarray.

^d^ Adjusted hazard ratio.

^e^ Cox proportional hazards model.

^f^ These calculations were obtained in the multivariate analysis performed with *LDHA*. The values of FIGO obtained in the multivariate analysis with the other markers are not shown but are similar to these values.

^g^ Univariate analysis was performed considering one variable for the analysis.

^h^ Multivariate analysis was performed considering gene expression and FIGO stage for the analysis.

### 3.3 Validation of LDHA and PFKP expression at the mRNA and protein levels

**FIGURE 4:**
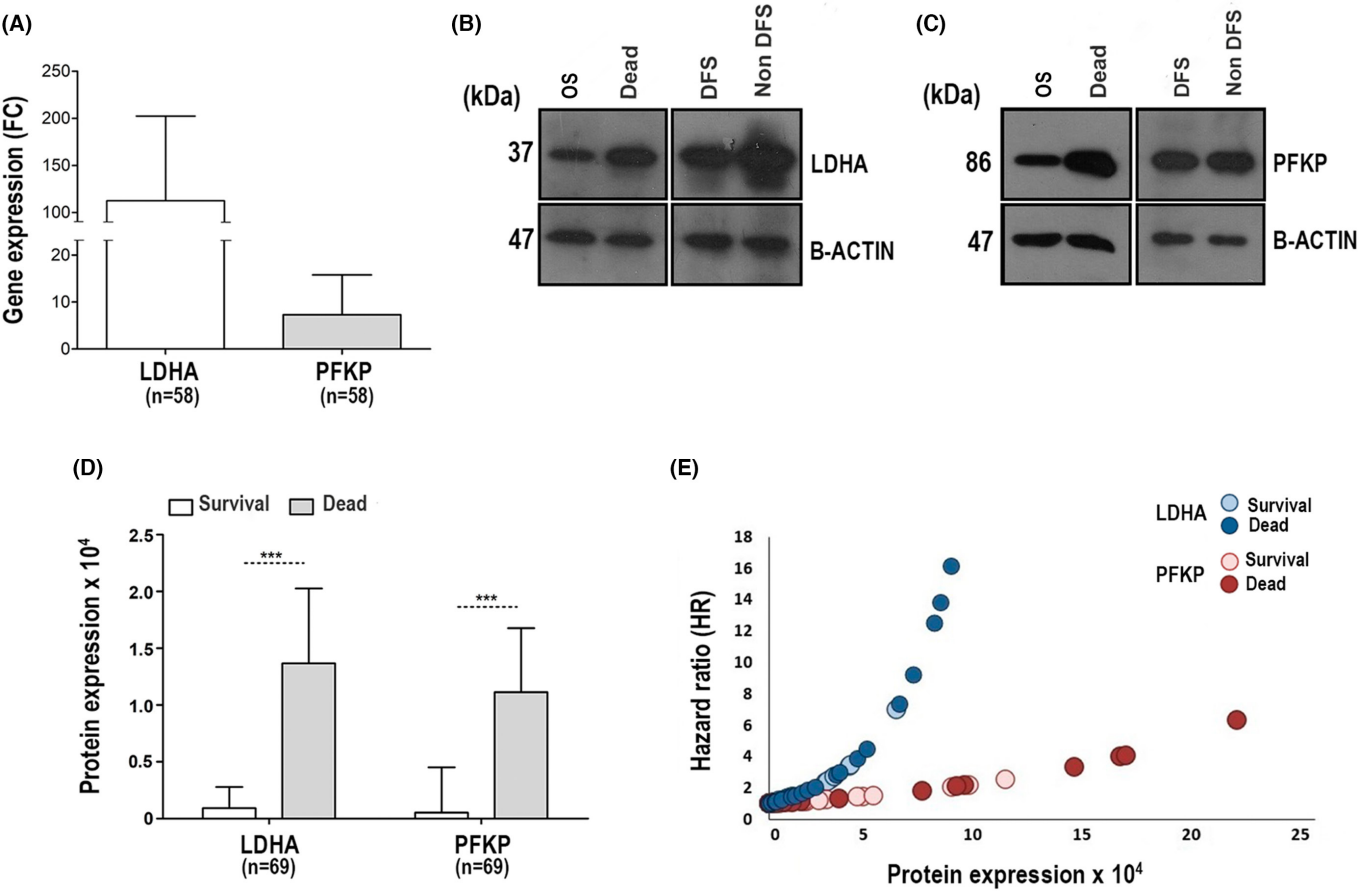
Validation of LDHA and PFKP expression in CC. The expression of LDHA and PFKP in CC was validated at the mRNA and protein levels by qRT–PCR and WB, respectively. Panel A shows the qRT–PCR analysis of *LDHA* and *PFKP* mRNA in 58 CCs. The expression was normalized with respect to the internal control (*RPS13*) and the control group by the double delta method using the final formula (2^−ΔΔCT^). Panels B and C show representative images of WB of LDHA and PFKP expression, respectively. OS = overall survival, DFS = disease‐free survival, non‐DFS = patients who died or surviving with the disease. The molecular weight of the proteins is shown in kilodaltons (kDa). The protein β‐actin was used as an internal control. All samples were derived from the same experiment, and gels and blots were processed in parallel. Panel D shows the mean expression ± SD of LDHA and PFKP proteins between patients with CC who survived (white bars, *n* = 47) and those who died (gray bars, *n* = 22). The intensity of LDHA and PFKP was normalized with respect to β‐actin. The expression is shown as optical density (OD) units. The significant differences between the groups were calculated with the Mann–Whitney U test, and *p* < 0.05 was considered statistically significant. Panel E shows the hazard ratio (HR) analysis in relation to LDHA and PFKP protein expression in CC. The risk of death from CC increases exponentially as protein expression (OD) increases, but it is more evident with the expression of LDHA (dark blue circles represent dead patients, while light blue circles represent surviving patients) than PFKP (dark red circles represent dead patients, while light red circles represent surviving patients). SD = standard deviation.

The expression of the *LDHA* and *PFKP* genes was validated by qRT–PCR, WB, and IHC. qRT–PCR confirmed that the expression of both genes was higher in the invading CCs (*n* = 58) than in HCTs (*n* = 19). However, the difference was much greater for *LDHA* (FC = 100.3; *p* = 9.8 × 10^−8^, Mann–Whitney test) than for *PFKP* (FC = 4.3, *p* = 2.0 × 10^−6^, Mann–Whitney test, Figure 4A). In addition, the expression of both genes was similar between CC HPV16+ and CC other‐HPVs+ [(*LDHA* FC =112.8 vs. 72.9, respectively *p* = 5.4 × 10^−1^, Mann–Whitney test); (*PFKP* FC = 5.6 vs. 3.9, respectively, *p* = 1.6 × 10^−1^, Mann–Whitney test)]. Interestingly, we found that the expression levels of *LDHA* and *PFKP* were on average 1.6 and 1.7 times higher, respectively, in patients who died or survived with the disease than in those who survived and were cured (*p* = 9.0 × 10^−3^ to *p* = 2.8 × 10^−2^; Mann–Whitney test, see Table S6).

In addition, we confirmed the presence of LDHA and PFKP proteins by WB in 69 CC samples. LDHA and PFKP proteins were expressed at higher levels in the tumors of patients who died (FC = 14.9, *p* = 3.0 × 10^−3^ and FC = 21.4, *p* = 1.8 × 10^−3^, respectively; Mann–Whitney test) or survived with the disease (FC = 29.1, *p* = 1.4 × 10^−4^ and FC = 17.2, *p* = 1.7 × 10^−3^, respectively; Mann–Whitney test, Figure 4B–D) compared to tumors of patients who survived free of the disease for more than 5 years.

**FIGURE 5:**
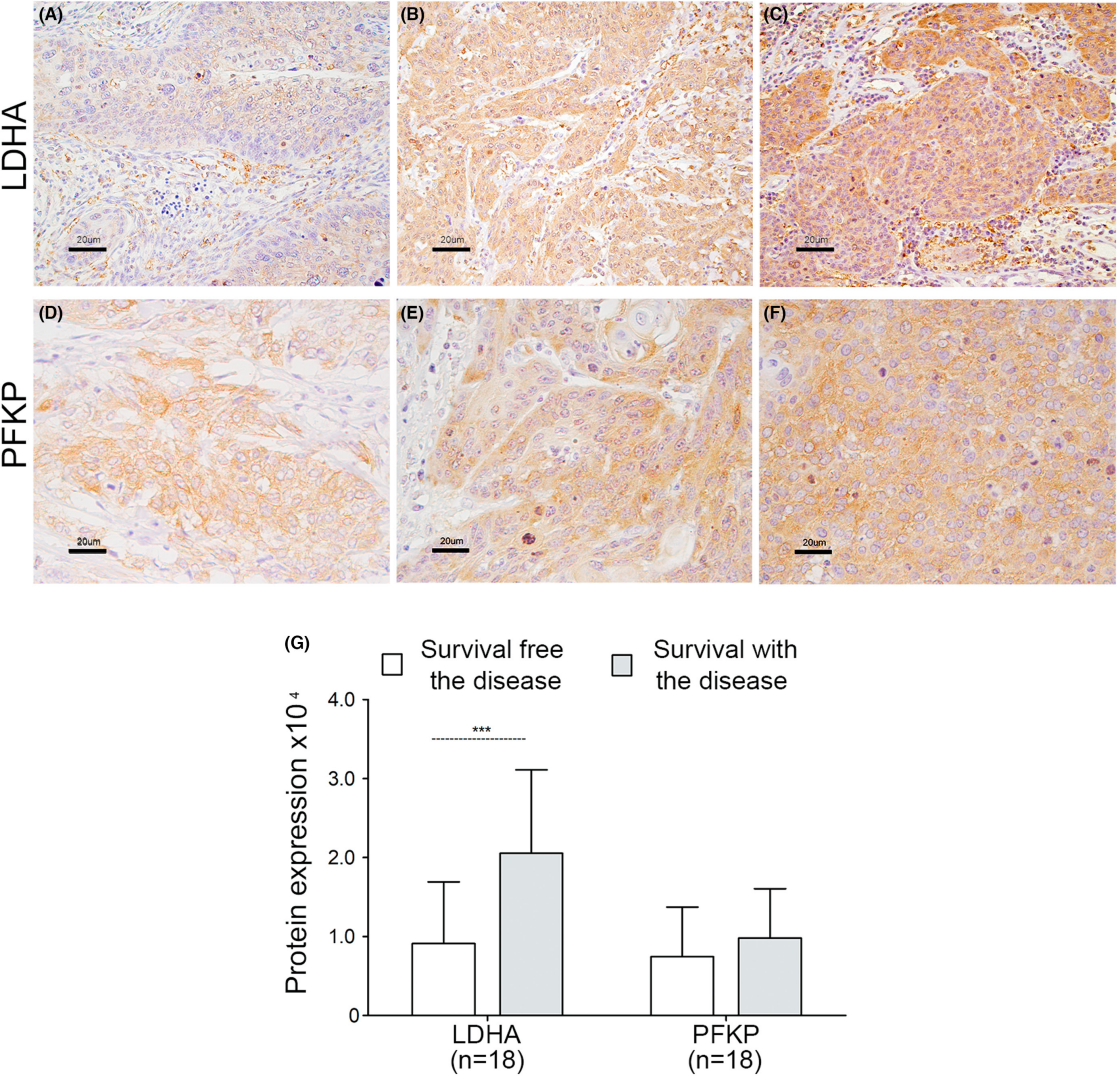
Expression of LDHA and PFKP proteins according to IHC. The expression of the LDHA and PFKP proteins was determined by IHC. Histological analysis included 18 CC and 6 metastatic tissues. A representative image of the experiments is shown. The detection of LDHA protein (panels A–C) and PFKP protein (panels D–F) was performed with specific antibodies. Panels A and D show the detection of LDHA and PFKP, respectively, in CC tissues of patients with DFS. Panels B and E show the expression of LDHA and PFKP, respectively, in CC tissues of patients who surviving with the disease. Panels C and F show the detection of LDHA and PFKP, respectively, in metastatic tissue from patients who surviving with the disease. The specific signal for proteins is shown in brown color and counterstained with hematoxylin in violet color. Original magnification 400x; the bars represent 20 μm. Panel G shows the quantitative analysis of LDHA and PFKP expression in CC tissues of patients with disease‐free survival (*n* = 8) versus patients who surviving with the disease (*n* = 10). The average optical density and staining area of LDHA and PFKP (DOI) in the tissues were considered. The mean ± SD of three independent experiments is shown. The Mann–Whitney test was performed to assess the difference between the groups, and *p* < 0.05 was considered to indicate statistical significance. SD = standard deviation.

Additionally, we analyzed the expression of these glycolytic enzymes by IHC in HCTs (*n* = 12), CC tissues (*n* = 18), and metastatic tissues (*n* = 6) preserved in paraffin from a new group of patients. LDHA and PFKP expression levels were significantly higher in tumor tissues than in HCTs (FC = 4.3 for LDHA and FC = 27.2 for PFKP); interestingly, the expression levels of both proteins were even higher in CC metastases than in HCTs (FC = 10.4 and 42.7, respectively) (see Table S7). This suggests that overexpression of LDHA and PFKP could be an important factor not only for tumor progression but also for the development of metastases. Interestingly, we reconfirmed that LDHA expression was higher in patients who died or survived with the disease than in cured patients (see Figure 5A–C,G). In contrast, there were no statistically significant differences in PFKP expression between the groups (see Figure 5D–F,G).

### 3.4 The *LDHA* and *PFKP* mRNA and protein expression are good markers of survival in CC

**FIGURE 6:**
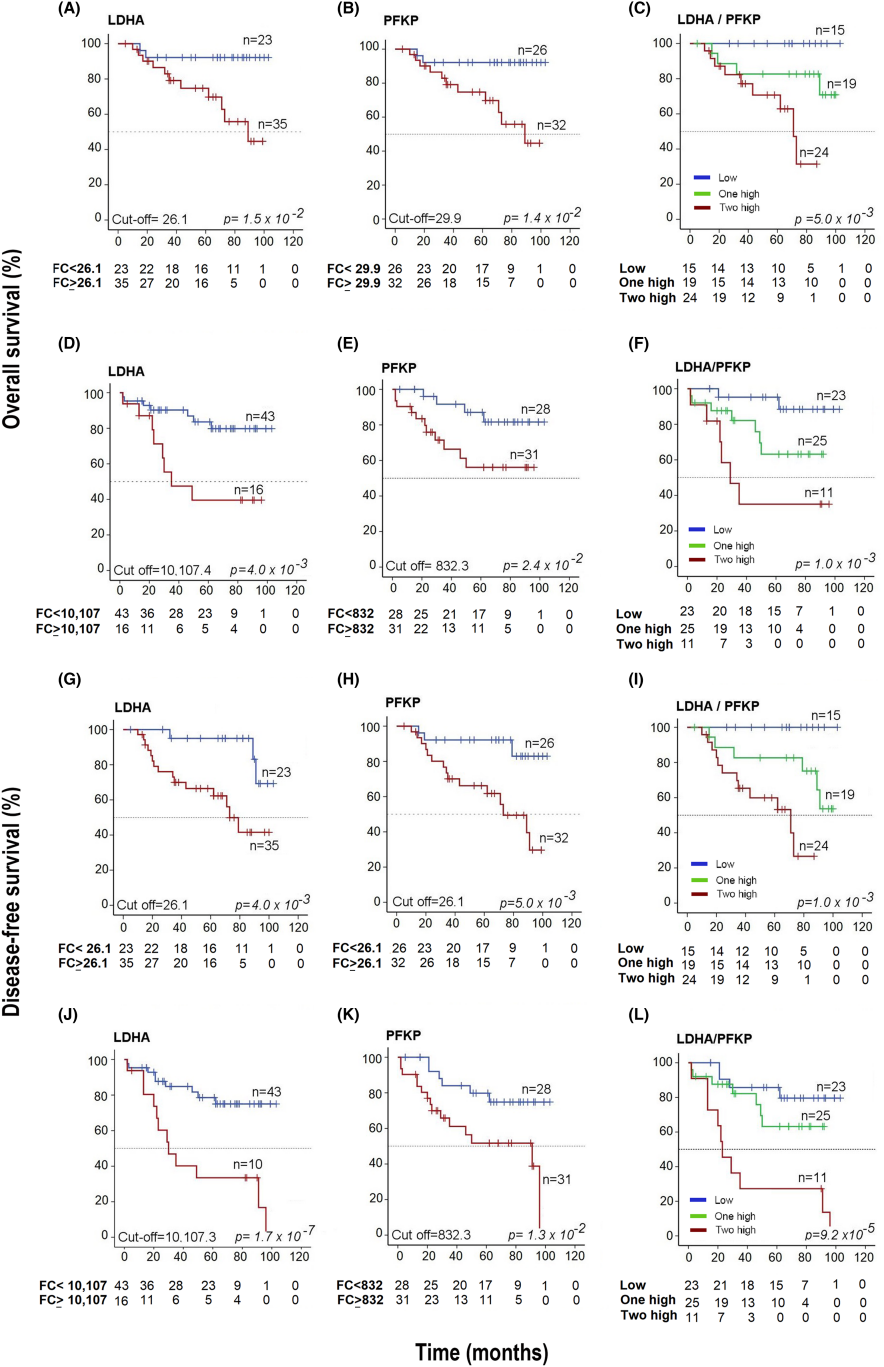
Kaplan–Meier survival curves based on LDHA and PFKP expression. OS analysis according to the expression of LDHA and PFKP by qRT–PCR (panels A–C) and WB (panels D–F). DFS analysis according to the expression of LDHA and PFKP by qRT–PCR (panels G–I) and WB (panels J–L). The cutoff values were calculated using ROC curves. In the OS analysis, the red lines include the values of patients with overexpression of LDHA or PFKP, and the blue line includes the values of patients without overexpression of LDHA or PFKP (panels A, B, D, and E, respectively). In panels C and F, the red line includes the values of patients with overexpression of two markers (LDHA/PFKP), the green line includes the values of patients with overexpression of only one marker (LDHA or PFKP), and the blue line includes the values of patients when neither of these two markers was overexpressed. In the DFS analysis, the red lines contain the values of patients with LDHA or PFKP overexpression, while the blue lines include the values of patients without LDHA or PFKP overexpression (panels G, H, J, and K, respectively). In panels I and L, the red line includes the values of patients with overexpression of two markers (LDHA/PFKP), the green line includes the values of patients with overexpression of only one marker (LDHA or PFKP), and the blue line includes the values of patients when neither of these two markers was overexpressed. Censored patients are shown marked with vertical bars. The number of patients at risk in each time intervals are noted in the tables below the curves. The *p value* was calculated with the log‐rank test.

At both the mRNA (*n* = 58 CC) and protein (*n* = 69) levels, we confirmed that the overexpression of LDHA and PFKP was associated with a significant decrease in OS and DFS during more than 5 years of follow‐up; however, the results were stronger when the protein levels were used for analysis. The analysis of mRNA is shown in Table S8 and Figure 6A–C,G–I.

At the protein level, we found that the OS rate was decreased markedly in the LDHA or PFKP overexpression group compared with the group without overexpression: 39% versus 82% and 55% versus 83% (both *p* < 0.05, log‐rank test), respectively (see Figure 6D,E). Similar results were found when DFS was analyzed (Figure 6J,K). Interestingly, when both proteins were overexpressed (LDHA/PFKP), OS and DFS decreased dramatically to 29% and 23%, respectively; in contrast, when there was a single overexpressed protein, the OS rate was 64%, and when neither of these two proteins was expressed, the OS rate was 90% (*p* = 1.0 × 10^−3^ and *p* = 9.2 × 10^−5^, log‐rank test; see Figure 6F,L).

With the univariate Cox analysis, the risk of death was much higher in patients with advanced FIGO stages than in patients with overexpression of either of the two markers (Table 2). However, when both markers were overexpressed, they conferred a greater risk of death than FIGO [HR = 7 (95% CI 1.6–31.1, *p* = 1.0 × 10^−2^) versus HR = 8.1 (95% CI = 2.6–26.1; *p* = 4.3 × 10^−4^)]. Similar figures were seen for DFS (Table 2). Interestingly, in the multivariate analysis including clinical stage, both proteins remained together with clinical stage in the models and still predicted OS or DFS, indicating that they confer a risk of death independent of FIGO stage, even of similar magnitude or greater than that conferred by FIGO stage when both markers are overexpressed [OS: HR = 6.1 (95% CI = 1.3–31.2; *p* = 1.8 × 10^−2^) vs. HR = 6.6 (95% CI = 1.3–32.1; *p* = 2.5 × 10^−2^)] and [DFS: HR = 4.8 (95% CI = 1.3–17.8; *p* = 1.8 × 10^−2^) vs. HR = 5.1 (95% CI = 1.5–16.6; *p* = 7.0 × 10^−3^)]. In fact, the HR increased exponentially as the expression level of these markers, especially LDHA, increased (Figure 4E). In five patients, the HR was well above the average HR of four, reaching an HR value of 16.1 in the patient with an LDHA intensity of 83,538 OD units.

**TABLE 2:** Hazard ratio analyses for patients with CC based on Cox proportional hazards models including the expression of the glycolytic proteins LDHA and PFKP and FIGO clinical stage.

Covariates	*n*	Univariate analysis^e^			Multivariate analysis^f^		
		HR^c^	95% CI	*p* ^d^	HR^c^	95% CI	*p* ^d^
Overall survival							
LDHA							
FIGO stage<IIA^a^	27	1			1		
FIGO stage>IIB	32	7.0	1.6–31.3	1.0 × 10^−2^	5.5	1.2–25.2	2.7 × 10^−2^
Low^b^	43	1			1		
High	16	4	1.4–11.1	8.0 × 10^−3^	2.8	1.0–7.9	5.2 × 10^−2^
PFKP							
FIGO stage<IIA^a^	27	1			1		
FIGO stage>IIB	32	7.0	1.6–31.3	1.0 × 10^−2^	6.9	1.5–30.5	1.1 × 10^−2^
Low^b^	33	1			1		
High	26	3.3	1.1–10.55	4.0 × 10^−2^	3.2	1.0–10.2	4.6 × 10^−2^
LDHA/PFKP^g^							
FIGO stage<IIA^a^	27	1			1		
FIGO stage>IIB	32	7.0	1.6–31.3	1.0 × 10^−2^	6.1	1.3–31.2	1.8 × 10^−2^
Low^b^	23	1			1		
One high	25	2.2	0.65–7.6	2.0 × 10^−1^	5.3	1.0–25.6	4.0 × 10^−2^
Two high	11	8.1	2.6–26.10	4.3 × 10^−4^	6.6	1.3–32.1	2.5 × 10^−2^
Disease‐free survival							
LDHA							
FIGO stage<IIA^a^	27	1			1		
FIGO stage>IIB	32	6.4	1.9–21.7	3.0 × 10^−3^	4.6	1.3–16.2	1.7 × 10^−2^
Low^b^	43	1			1		
High	16	4.5	1.9–10.8	1.0 × 10^−3^	3.2	1.3–7.6	1.2 × 10^−2^
PFKP							
FIGO stage<IIA^a^	27	1			1		
FIGO stage>IIB	32	6.4	1.9–21.7	3.0 × 10^−3^	6.1	1.7–20.6	4.0 × 10^−2^
Low^b^	28	1			1		
High	31	3.2	1.2–8.2	1.8 × 10^−2^	2.9	1.1–7.5	2.8 × 10^−2^
LDHA/PFKP^g^							
FIGO stage<IIA^a^	27	1			1		
FIGO stage>IIB	32	6.4	1.9–21.7	3.0 × 10^−3^	4.8	1.3–17.9	1.8 × 10^−2^
Low^b^	23	1			1		
One high	25	2.2	0.7–7.6	2.0 × 10^−1^	2.7	0.8–9.5	1.1 × 10^−1^
Two high	11	8.1	2.5–26.1	4.3 × 10^−4^	5.1	1.5–16.6	7.0 × 10^−3^

Abbreviations: CI, confidence interval; FIGO stage, International Federation of Gynecology and Obstetrics stage; HR, hazard ratio.

^a^ FIGO stage analysis.

^b^ Optimal cutoff values were selected according to the ROC analysis in relation to the expression of LDHA or PFKP obtained with WB.

^c^ Adjusted hazard ratio.

^d^ Cox proportional hazards model.

^e^ Univariate analysis was performed considering one variable for the analysis.

^f^ Multivariate analysis was performed considering gene expression and FIGO stage for the analysis.

^g^ Low = downregulation of two genes; one high = upregulation of one gene; two high = upregulation of LDHA and PFKP.

### 3.5 Analysis of gene expression with data from the TCGA database

Additionally, we explored the glycolysis gene expression profile, with 12 of the 14 genes studied in our samples, in 295 CC and 3 HCT samples from the TCGA database. The distribution of the CCs and HCTs according to the expression profiles in the hierarchical grouping was very similar to the distribution of the samples explored in our work (see Figure S6). Although there was a significant difference in the distribution of CCs according to the FIGO stage (≤IIA versus ≥IIB; *p* = 1.2 × 10^−3^, Table S2) and gene profile. On the other hand, as in our samples, the level of expression of the *LDHA* and *PFKP* genes was not different, or the difference was marginal, between the two FIGO groups (Figure S7).

Remarkably, we confirmed that the overexpression of *LDHA* and *PFKP* genes was associated with a significant decrease in the OS (from 54% to 28% for *LDHA* overexpression and from 52% to 35% for *PFKP* overexpression; *p ≤ 5.5* × *10* ^*−4*^, log‐rank test), and the difference was more profound when both genes were overexpressed (the OS rate decreased up to 25%; *p* = 1.2 × 10^−4^, log‐rank test; Figure S7); overexpression of these genes was associated with an increased risk of death (HR = 3.2, 95% CI 1.7–6.1, *p* = 3.3 × 10^−4^) regardless of the FIGO clinical stage (Table S9).

## 4 DISCUSSION

This is the first study in which it was identified that overexpression of the glycolysis pathway genes *LDHA* and *PFKP*, both at the mRNA level and protein level, is a good prognostic marker for OS and DFS in patients with CC, independent of FIGO stage. In fact, the risk of death when these two markers are elevated is equal to or greater than that of advanced FIGO stage and increases exponentially along with the protein level in the tumor, especially for LDHA. These findings were confirmed in the analysis that we performed on 295 CCs included in the TCGA database.

LDHA is part of the enzyme lactate dehydrogenase (LDH), which converts pyruvate into lactate. This enzyme is composed of four subunits, which can be A (LDHA), B (LDHB), or a combination of both.^26^ Previous studies have shown that the isoforms in which the A subunit predominates favor the conversion of pyruvate into lactate, which stimulates glycolysis instead of oxidative phosphorylation. In contrast, when the B subunit predominates, the reverse happens: lactate is converted to pyruvate and metabolized by the Krebs/oxidative phosphorylation cycle.^27^ In this work, we show that in CC, subunit A is overexpressed, which indicates that LDHA favors the production of lactate and thus anaerobic metabolism, which can provide growth advantages to CC. On the other hand, PFKP, an isoform of the enzyme phosphofructokinase 1 (PFK‐1), stimulates the activity of glycolysis by catalyzing the formation of fructose 1,6‐bisphosphate from fructose 6‐phosphate, the first rate‐limiting step of glycolysis, and consequently the production of pyruvate. The simultaneous overexpression of PFKP and LDHA benefits the tumor because the concerted action of the two enzymes in CC could facilitate rapid conversion of pyruvate to lactate, accelerating glycolysis (10–100 times faster than total glucose oxidation in the mitochondria) and generating a large amount of ATP via anaerobic mechanisms. This facilitates tumor growth and the development of more aggressive invasive tumors.^10^

No studies have assessed the influence of *LDHA* gene expression on CC aggressiveness and the survival of patients. A few studies have focused on *LDHA* as a part of the tumor gene expression profile associated with metastasis^28^ and resistance to chemotherapy^29^; however, contradictory results have been reported. *LDHA* upregulation was associated with resistance to chemotherapy,^29^ *LDHA* downregulation was associated with tumor metastases.^28^ Interestingly, in this last study, tumors with FIGO stage ≤IIB predominated, while in the first study, tumors with FIGO stage ≥IIB predominated. This could suggest that large, advanced‐stage tumors likely already exhibit hypoxia, so anaerobic metabolism predominates, while in smaller, early‐stage tumors, aerobic metabolism predominates, as was also observed in the TCGA samples analyzed in this work.

On the other hand, several studies have shown that increased serum LDH activity in patients with CC is associated with a poor prognosis and decreased OS^30^ and DFS^31^; it is also associated with an increased risk of death or recurrence independent of other clinical factors.^32^ However, the limitation of these studies was that they did not demonstrate whether LDH levels were quantified specifically in CC tissues or other tissues, as this enzyme is produced in several tissues.

In several types of tumors (such as squamous cell carcinoma of the skin and melanoma), increased expression of glycolysis‐related genes is associated with tumor progression and decreased survival time in patients.^33^ In addition, in many types of cancers, it has been observed through PET using fluorodeoxyglucose (FDG) that increased tumor glucose consumption is related to tumor aggressiveness.^34^ This phenomenon has also been demonstrated in animal models. For example, in two mouse models of triple‐negative breast cancer (TNBC), 4T1 and Py8119, inhibition of glycolysis resulted in reduced tumor growth and metastases, which prolonged mouse survival.^35^ In CC cell lines, *LDHA* silencing has been shown to decrease some neoplastic features in vitro. For example, HeLa and SiHa cells exhibited decreased colony formation and invasion capacity when the gene was silenced by miR‐34a. Interestingly, when the activity of miRNA was finished, the activity of *LDHA* was restored to baseline levels, favoring cell proliferation and invasion, demonstrating the importance of the expression of this gene for the tumor neoplastic phenotype.^36^

Considering the importance of the neoplastic phenotype and tumor metabolism, LDHA could be a promising therapeutic target in CC. Several pharmacological inhibitors for LDHA have previously been reported for use in cancer, and there are currently several studies looking for more selective inhibitors.^37^, ^38^ One of these compounds, gossypol, is being used in clinical trials for the treatment of malignant glioma (NCT00540722 and NCT00390403).

Although there are no reports on PFKP in CC, this enzyme has been found to be overexpressed in HeLa cells^39^ and related to the activation of tumor survival pathways via P44/42 mitogen‐activated protein kinase (MAPK).^40^ Increased PFKP expression and activity are related to neoplastic activity, metastasis, and decreased survival in several types of cancer, primarily brain, kidney, and breast cancers.^41^, ^42^ Other studies have shown that the inhibition of *PFKP* with specific siRNAs in lung cancer cell lines^37^ and murine tumor models of leukemia^43^ decreased the expression of the enzyme, the glycolysis rate, and glucose, lactic acid, and ATP concentrations in the supernatant of cell cultures; tumor growth, and progression was also observed.

Simultaneous overexpression of *PFKP* and *LDHA* has previously been described in a breast cancer cell line (MDA‐MB‐231), in which *PFKP* regulation also affects lactate production. Interestingly, quercetin treatment impaired the PFKP‐LDHA signaling axis, thereby inhibiting anaerobic glycolysis, cell migration, and cell invasion in vitro by 80%,^42^ demonstrating that inhibition of both enzymes may be useful in the treatment of cancers in which these enzymes are activated, such as CC.

## 5 CONCLUSIONS

The overexpression of the glycolytic enzymes LDHA and PFKP at the mRNA and protein levels was associated with poor overall and disease‐free survival in CC. Overexpression of LDHA and PFKP increased the risk of death from CC by eightfold, and this effect was independent of the FIGO clinical stage. In fact, the risk of death from CC increased exponentially as the expression level of these markers, mainly LDHA, increased. The measurement of the mRNA and protein levels of these two markers could be very useful to evaluate the clinical evolution and the risk of death from CC and to make better therapeutic decisions at the beginning of treatment.

## AUTHOR CONTRIBUTIONS

**Verónica Bolaños‐Suárez:** Conceptualization (lead); formal analysis (lead); investigation (lead); writing – original draft (lead); writing – review and editing (lead). **Ana Alfaro:** Investigation (equal). **Ana María Espinosa:** Investigation (equal). **Ingrid Medina‐Martínez:** Investigation (equal). **Eligia Juárez:** Methodology (equal). **Nicolás Villegas‐Sepúlveda:** Methodology (equal). **Marco Gudiño‐Zayas:** Data curation (equal). **América Gutiérrez‐Castro:** Investigation (equal). **Edgar Román‐Bassaure:** Methodology (equal). **María Eugenia Salinas‐Nieves:** Methodology (equal). **Sergio Bruno‐Muñoz:** Methodology (equal). **Carlos Aranda:** Methodology (equal). **Oscar Flores‐Herrera:** Methodology (equal). **Jaime Berumen:** Conceptualization (lead); formal analysis (lead); investigation (lead); writing – original draft (lead); writing – review and editing (lead).

## FUNDING INFORMATION

This research was conducted with support from the National Council on Science and Technology (Conacyt) under grant numbers 8135/A1, 24341 (to JB) and Laboratory Huella Génica.

## CONFLICT OF INTEREST STATEMENT

The authors declare that they have no competing interests or personal relationships that could have appeared to influence the work reported in this paper.

## ETHICS APPROVAL AND CONSENT TO PARTICIPATE

The study protocol was approved by the Scientific and Ethics Committee of the General Hospital of Mexico (HGM) (approval number DIC/03/311/04/051). All experiments and analyses in this study were performed in accordance with the Declaration of Helsinki.^18^ Written informed consent was obtained from all participants before their inclusion in the study.

## Supporting information


Figure S1.

Figure S2.

Figure S3.

Figure S4.

Figure S5.

Figure S6.

Figure S7.

Table S1.

## Data Availability

The datasets GSE52904 and GSE39001 analyzed during the current study are available in the GEO database (https://www.ncbi.nlm.nih.gov/geo/) and the NCBI‐GEO repository (https://www.ncbi.nlm.nih.gov/geo/query/acc.cgi?acc=GSE52904, https://www.ncbi.nlm.nih.gov/geo/query/acc.cgi?acc=GSE39001).
