# Supplementary figures and images for: The mRNA and protein levels of the glycolytic enzymes lactate dehydrogenase A (LDHA) and phosphofructokinase platelet (PFKP) are good predictors of survival time, recurrence, and risk of death in cervical cancer patients
Source: Cancer Med. 2023 Jun 16;12(14):15632–49. doi: 10.1002/cam4.6123 (PMC10417302; doi:10.1002/cam4.6123)

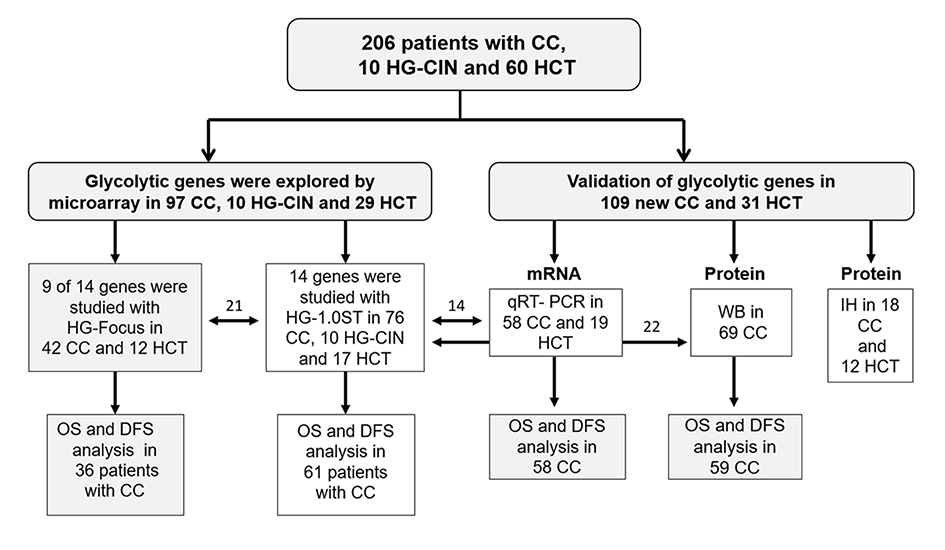

Supplement: Supplementary file 1 — Figure S1. [file CAM4-12-15632-s004.tif]

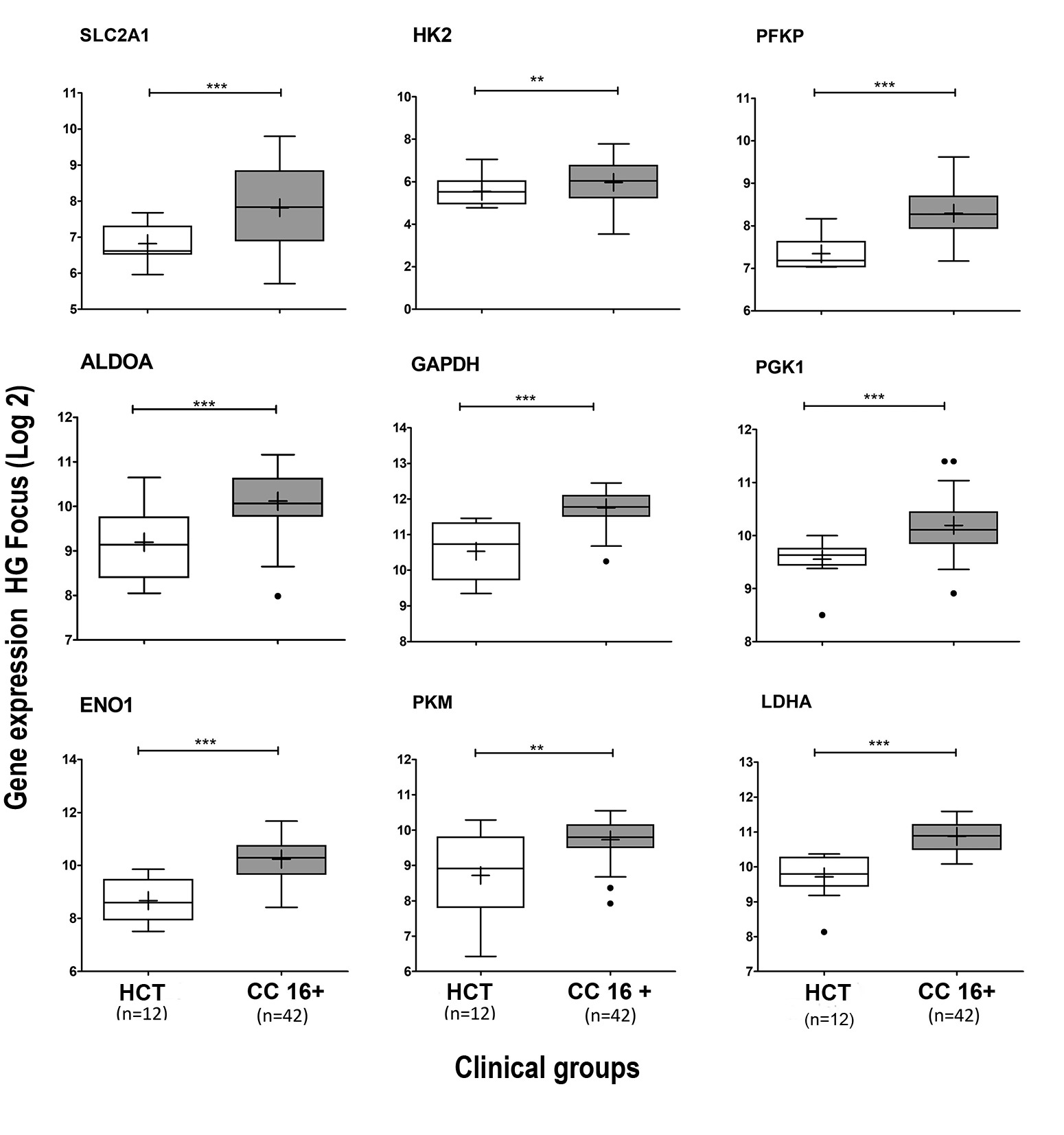

Supplement: Supplementary file 2 — Figure S2. [file CAM4-12-15632-s006.tif]

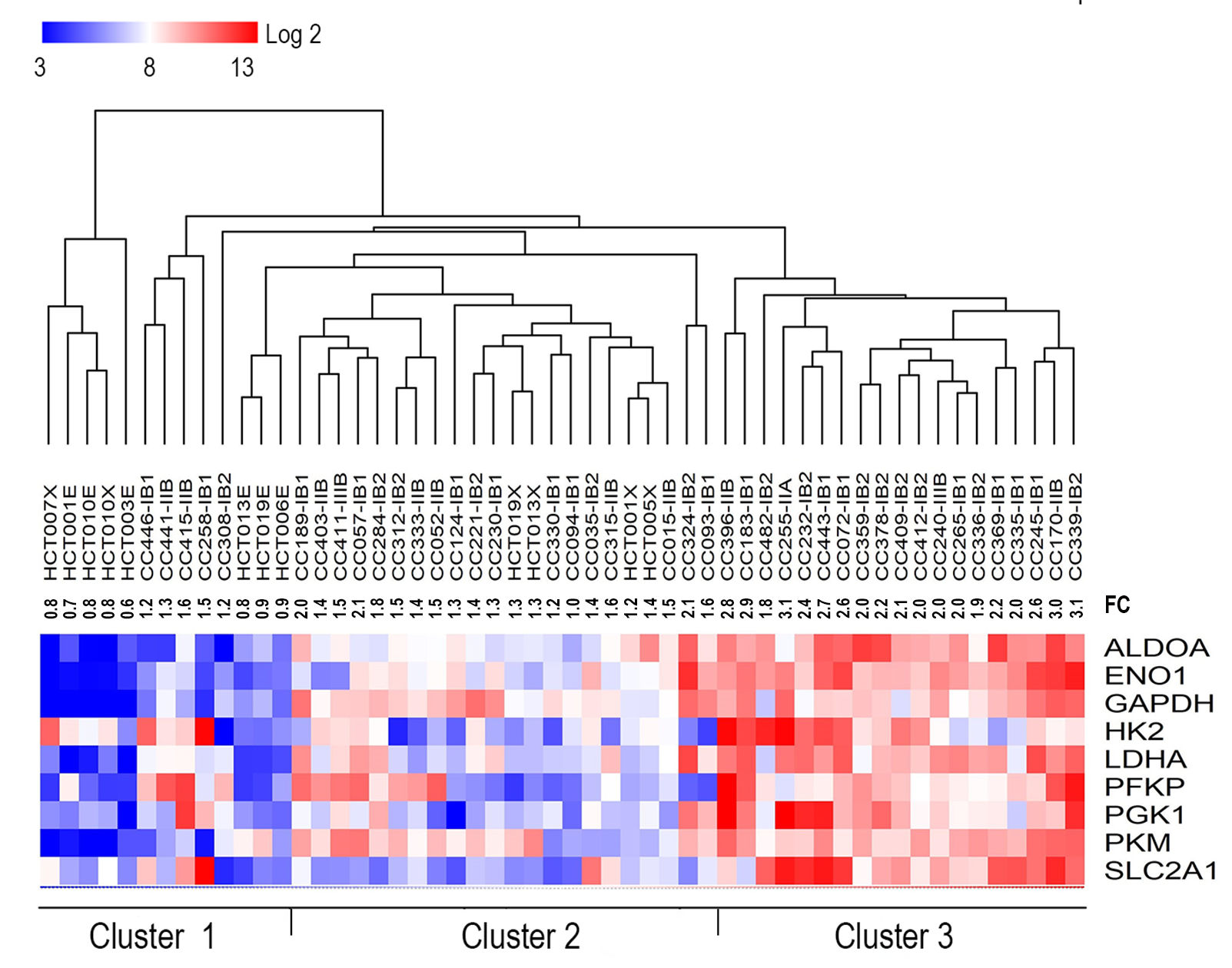

Supplement: Supplementary file 3 — Figure S3. [file CAM4-12-15632-s003.tif]

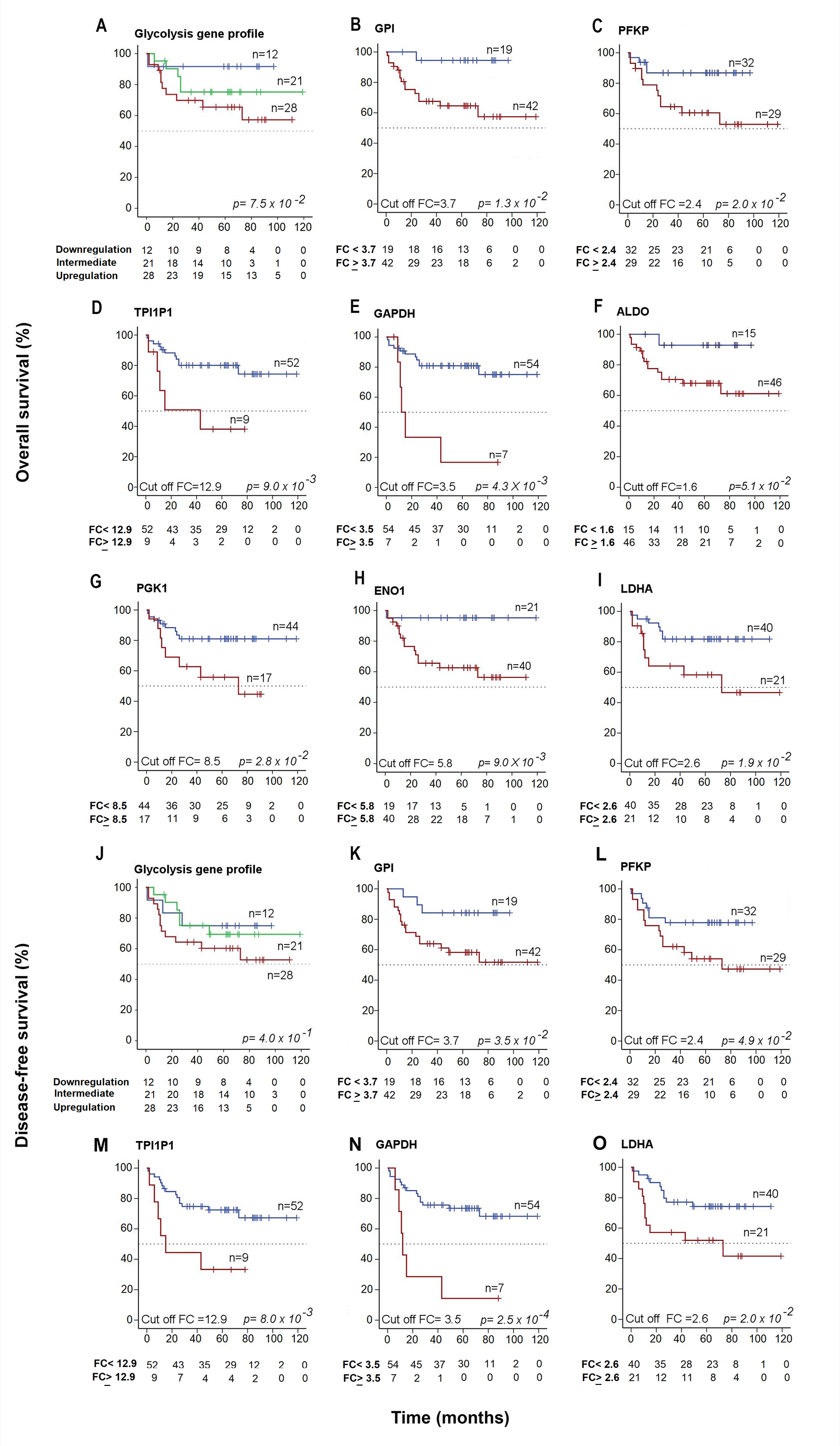

Supplement: Supplementary file 4 — Figure S4. [file CAM4-12-15632-s008.tif]

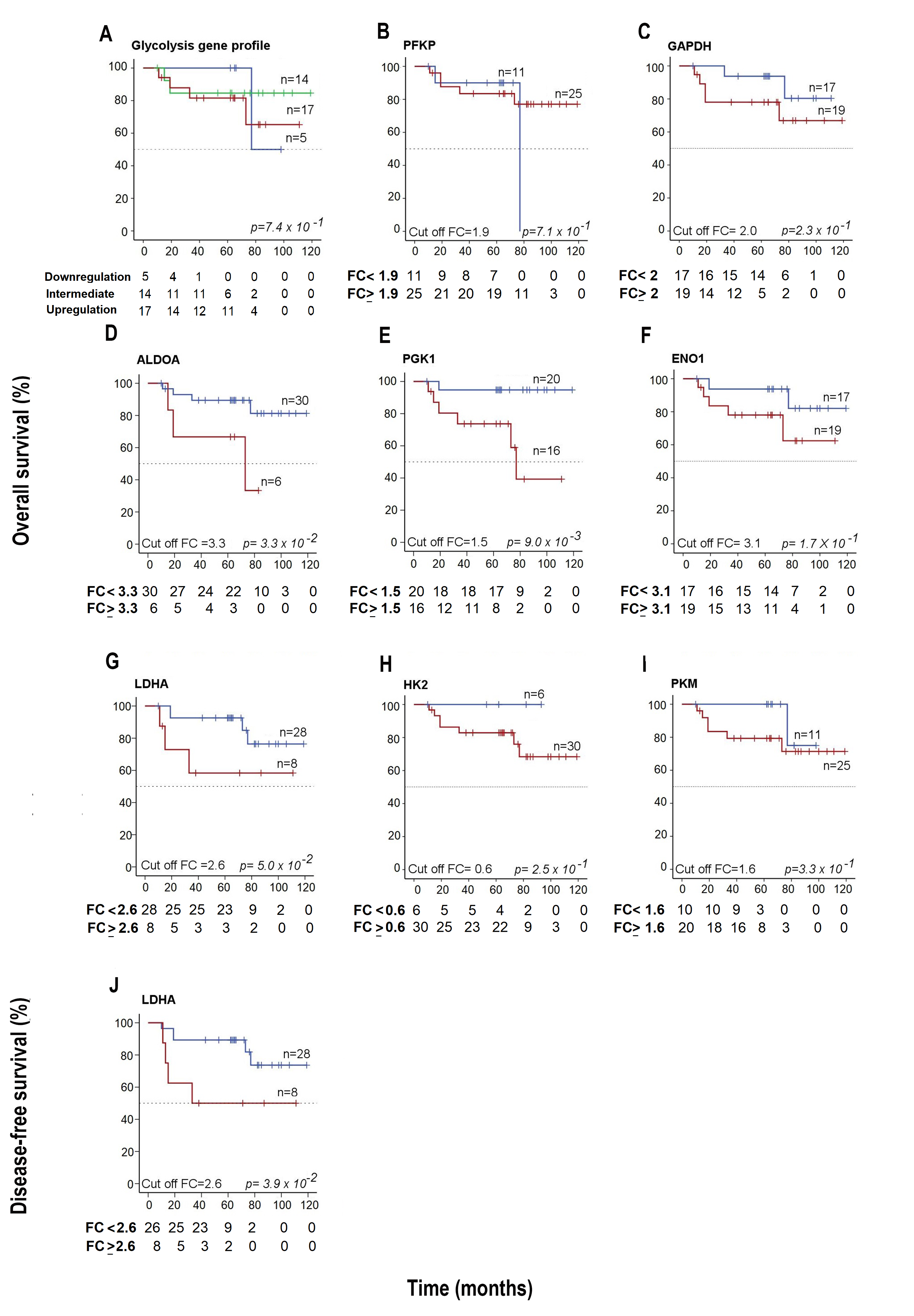

Supplement: Supplementary file 5 — Figure S5. [file CAM4-12-15632-s002.tif]

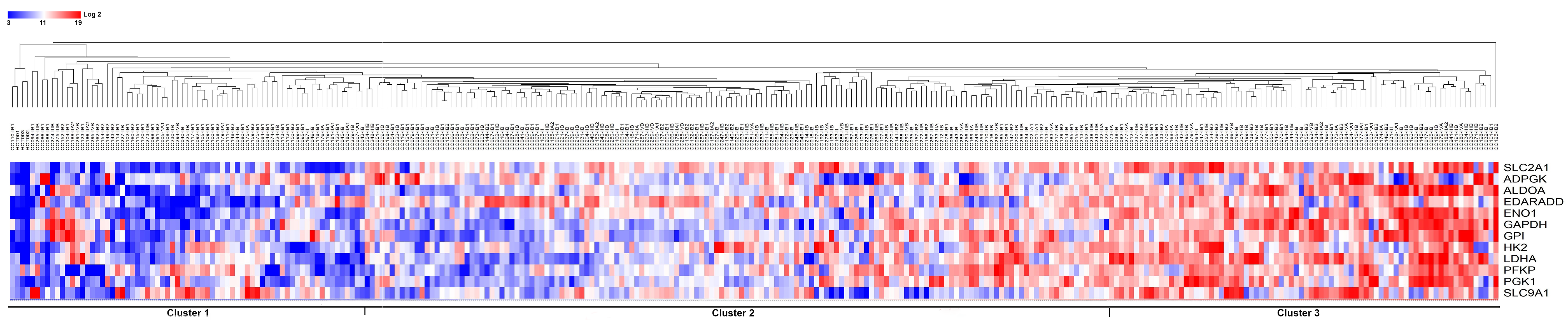

Supplement: Supplementary file 6 — Figure S6. [file CAM4-12-15632-s007.tif]

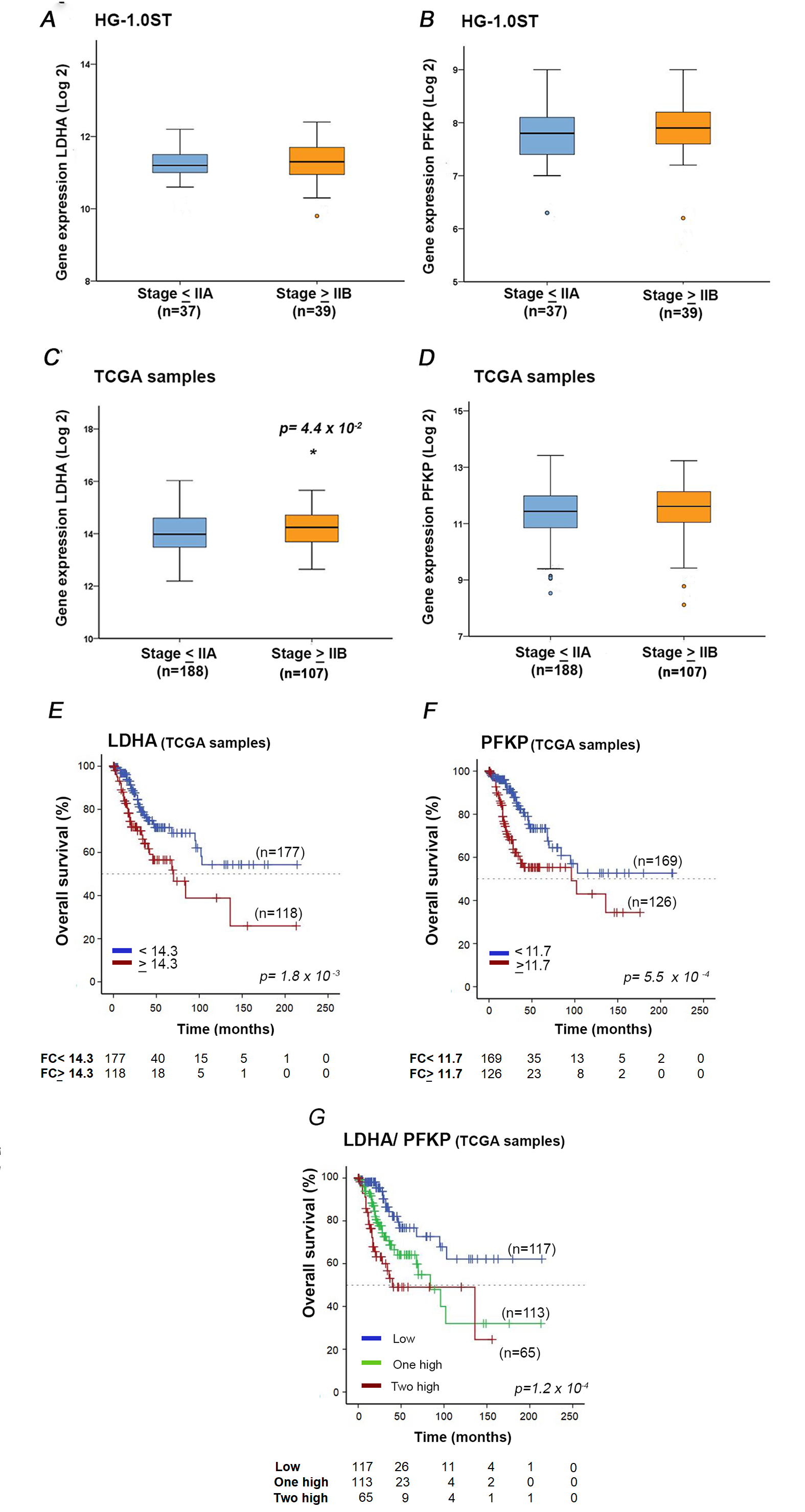

Supplement: Supplementary file 7 — Figure S7. [file CAM4-12-15632-s005.tif]
