# Supplementary material for: The mRNA and protein levels of the glycolytic enzymes lactate dehydrogenase A (LDHA) and phosphofructokinase platelet (PFKP) are good predictors of survival time, recurrence, and risk of death in cervical cancer patients
Source: Cancer Med. 2023 Jun 16;12(14):15632–49. doi: 10.1002/cam4.6123 (PMC10417302; doi:10.1002/cam4.6123)
Supplement: Supplementary file 8 — Table S1. [file CAM4-12-15632-s001.docx]

**Supplementary Table 1.** Clinicopathological characteristics

of 206 patients with cervical cancer^a^.

| **Variables** |  |
| --- | --- |
| Age, mean ± SD | 51±14 |
| FIGO stage, n (%) |  |
| ≤IA2 | 5 (2.4) |
| IB1 | 67 (32.5) |
| IB2 | 26 (12.6) |
| IIA | 10 (4.9) |
| IIB | 56 (27.2) |
| IIIA | 2 (1) |
| IIIB | 27 (13.1) |
| ≥IV | 13 (6.3) |
| Tumor Histology, n (%) |  |
| ACC | 36 (17.4) |
| ASCC | 4 (2) |
| SCC | 166 (80.6) |
| Viral type, n (%) |  |
| HPV16+ | 132 (64.1) |
| Other HPVs+^b^ | 56 (27.2) |
| Unknown HPV | 18 (8.7) |
| Recurrence, n (%) |  |
| No | 138 (67) |
| Yes | 68 (33) |
| Mortality, n (%) |  |
| No | 148 (71.8) |
| Yes | 58 (28.2) |

ACC= Adenocarcinoma; ASCC= Adenosquamous Cell Carcinoma; SCC=Squamous

Cell Carcinoma; SD= standard deviation.

1. All patients received complete clinical evaluation and were treated with surgery,

radiation, chemotherapy, or a combination of these according to American

Cancer Society guidelines.

b. Positive for HPVs 11, 18, 31, 33, 35, 42, 45, 51, 52, 53, 58, 59, 64 and 68.

**Supplementary Table 2.** Segregation of HCT, HG-CINs, CCs and cell lines according to glycolytic

gene expression profile in the hierarchical analysis.

| **Clinical groups** | **n** | **Downregulation** | **Intermediate regulation** | **Upregulation** | ***p value^a^*** |
| --- | --- | --- | --- | --- | --- |
|  |  | Cluster 1 | Cluster 2 | Cluster 3 |  |
| **HG-1.0ST** |  |  |  |  |  |
| HCT | 17 | 13 (76.5) | 4 (23.5) | 0 |  |
| HG-CINs | 10 | 4 (40.0) | 6 (60.0) | 0 | *5.8 x 10^-1b^* |
| Cell lines | 3 | 0 | 0 | 3 (100) | *3.4 x 10^-2b^* |
| CC^d^ | 76 | 15 (19.7) | 33 (43.5) | 28 (36.8) | *5.2 x 10^-4b^* |
| CC ≤IIA^e^ | 37 | 9 (24.3) | 18 (48.6) | 10 (27.1) | *2.1 x 10 ^-1c^* |
| CC ≥IIB | 39 | 6 (15.4) | 15 (10.3) | 18 (46.1) |  |
| CC HPV16+ | 57 | 9 (15.8) | 24 (42.1) | 24 (42.1) | *1.6 x 10^-1d^* |
| CC HPVs+ | 19 | 6 (31.6) | 9 (47.3) | 4 (21.1) |  |
| **HG-Focus** |  |  |  |  |  |
| HCT | 12 | 8 (66.7) | 4 (33.3) | 0 | *6.9 x 10^-4b^* |
| CC HPV16+ | 42 | 5 (11.9) | 18 (42.9) | 19 (45.2) |  |
| CC ≤IIA^e^ | 31 | 3 (9.7) | 12 (38.7) | 16 (51.6) | *3.6 x 10^-1c^* |
| CC ≥IIB^f^ | 11 | 2 (18.2) | 6 (54.5) | 3 (27.3) |  |
| **HG-1.0ST + HG-Focus** | | | | |  |
| CC ≤IIA | 68 | 12 (17.7) | 30 (44.1) | 26 (38.2) | *9.1 x 10^-1c^* |
| CC ≥IIB | 50 | 8 (16) | 21 (42) | 21 (42) |  |
| **TCGA data** | | | | |  |
| HCT | 3 | 3 (100) | 0 | 0 |  |
| CC | 295 | 67 (22.7) | 148 (50.2) | 80 (27.1) |  |
| CC ≤IIA | 188 | 56 (29.8) | 86 (45.7) | 46 (24.5) | *1.2 x 10^-3c^* |
| CC ≥IIB | 107 | 12 (11.2) | 64 (59.8) | 31 (29) |  |

CC= cervical cancer; HG-CINs= high-grade cervical intraepithelial neoplasms; HCT= healthy cervical tissue.

1. The *p value* was calculated with Chi-square test n x n. b. The *p value* was calculated with respect to the HCT group. c. The *p value*

was calculated with respect to the ≤IIA group. d. The *p value* was calculated with respect to the CC HPV16+ group. CC HPVs+ = HPVs 11,18, 31, 33, 35, 42, 45, 51, 52, 53, 58, 59, 64 and 68. e. CC ≤IIA= Figo stage IB1-IIA. f. CC ≥ IIB= FIGO stage.

**Supplementary Table 3**. Univariate and multivariate analyses of factoring affecting the DFS of patients with CC based on Cox proportional hazards models including the expression of glycolytic genes explored with the HG-1.0ST microarray and FIGO clinical stage.

| **Covariates** |  |  | **Univariate analysis^g^** | | |  | **Multivariate analysis^h^** | | |
| --- | --- | --- | --- | --- | --- | --- | --- | --- | --- |
|  | **n** |  | **HR^d^** | **95% CI** | **p^e^** |  | **HR^b^** | **95% CI** | **p^e^** |
| **FIGO** | | | | | | | | | |
| <IIA | 29 |  | 1.0 |  | |  | 1.0 |  | |
| >IIB | 32 |  | 3.7 | 1.3-10.0 | *1.2 x 10^-2^* |  | 3.6^d^ | 1.3-9.8^d^ | *1.3 x 10^-2f^* |
| **Glycolytic gene expression profile** | | | | | | | | | |
| FC < 1.98^a^ | 57 |  | 1.0 |  |  |  | 1.0 |  |  |
| FC ≥ 1.98 | 4 |  | 6.3 | 2.0-19.4 | *1.0 x 10 ^-3^* |  | 4.0 | 1.2-12.8 | *2.1 x 10 ^-2^* |
| Glycolysis FC score^b^ | 61 |  | 2.8 | 1.0-10.1 | *1.3 x 10 ^-1^* |  | 1.7 | 0.5-6.3 | *3.9 x 10 ^-1^* |
| ***LDHA*** | | | | | | | | | |
| Low^c^ | 40 |  | 1.0 |  | |  | 1.0 |  | |
| High | 21 |  | 2.7 | 1.1-6.3 | *2.6 x 10^-2^* |  | 2.7 | 1.1-6.2 | ***2.9 x 10^-2^*** |
| ***PFKP*** | | | | | | | | | |
| Low | 32 |  | 1.0 |  | |  | 1.0 |  | |
| High | 29 |  | 2.4 | 0.9-5.9 | *5.8 x 10^-2^* |  | 2.3 | 0.9-5.7 | *6.2 x 10^-2^* |
| ***GAPDH*** | | | | | | | | | |
| Low | 54 |  | 1.0 |  | |  | 1.0 |  | |
| High | 7 |  | 5.0 | 1.9-13.3 | *1.0 x 10^-3^* |  | 3.5 | 1.3-9.6 | *6.0 x 10^-2^* |
| ***TPI1P1*** | | | | | | | | | |
| Low | 52 |  | 1.0 |  | |  | 1.0 |  | |
| High | 9 |  | 3.3 | 1.3-8.6 | *1.3 x 10^-2^* |  | 2.2 | 0.8-5.9 | *1.3 x 10^-1^* |
| ***GPI*** | | | | | | | | | |
| Low | 19 |  | 1.0 |  | |  | 1.0 |  | |
| High | 42 |  | 3.4 | 1.0-11.64 | *4.9 x 10^-2^* |  | 3.2 | 0.9-10.7 | *6.5 x 10^-2^* |
| ***ENO*** | | | | | | | | | |
| Low | 21 |  | 1.0 |  | |  | 1.0 |  | |
| High | 40 |  | 2.6 | 0.8-7.8 | *8.1 x 10^-1^* |  | 1.9 | 0.6-6.1 | *2.2 x 10^-1^* |
| ***PGK1*** | | | | | | | | | |
| Low | 44 |  | 1.0 |  | |  | 1.0 |  | |
| High | 17 |  | 2.2 | 0.9-5.2 | *7.6 x 10^-1^* |  | 1.7 | 0.7-4.1 | *2.3 x 10^-1^* |
| ***ALDOA*** | | | | | | | | | |
| Low | 15 |  | 1.0 |  | |  | 1.0 |  | |
| High | 46 |  | 3.4 | 0.6-8.0 | *2.3 x 10^-1^* |  | 1.9 | 0.6-6.6 | *3.0 x 10^-1^* |

CI= confidence interval; HR= hazard ratio; FIGO stage=International Federation of Gynecology and Obstetrics stage.

a. Optimal cut off values were selected according to the ROC curve of the glycolysis FC score.

b. The analysis was performed considering the glycolysis FC score as continuous variable.

c. Optimal cutoff values were selected according to the ROC analysis in relation to the fold changes in gene expression obtained with the Human Gene 1.0ST microarray.

d. Adjusted hazard ratio.

e. Cox proportional hazards model.

f. These calculations were obtained in the multivariate analysis performed with *LDHA*. The values of FIGO obtained in the multivariate analysis with the other markers are not shown but are similar to these values.

g. Univariate analysis was performed considering one variable for the analysis.

h. Multivariate analysis was performed considering gene expression and FIGO stage for the analysis.

**Supplementary Table 4**. Univariate and multivariate analyses for the overall survival of patients with CC with Cox proportional hazards models including the expression of glycolytic genes explored with HG-Focus microarray and FIGO clinical stage.

| **Covariates** |  |  | **Univariate analysis^g^** | | |  | **Multivariate analysis^h^** | | | |
| --- | --- | --- | --- | --- | --- | --- | --- | --- | --- | --- |
|  | **n** |  | **HR^d^** | **95% CI** | **p^e^** |  | **HR^b^** | **95% CI** | **p^e^** | |
| **FIGO** | | | | | | | | | |  |
| <IIA | 26 |  | 1.0 |  | |  | 1.0 |  | | |
| >IIB | 10 |  | 3.1 | 1.0-9.5 | *5.0 X 10^-2^* |  | 3.0^d^ | 1.0-9.1^d^ | *5.0 X 10^-2f^* | |
| **Glycolytic gene expression profile** | | | | | | | | | |  |
| FC < 1.95^a^ | 18 |  | 1.0 |  |  |  | 1.0 |  |  | |
| FC ≥ 1.95 | 18 |  | 2.9 | 0.5-15.0 | *2.0 x 10 ^-1^* |  | 2.6 | 0.7-11.2 | *2.0 x 10^-1^* | |
| Glycolysis FC score^b^ | 36 |  | 1.3 | 0.4-4.6 | *7.0 x 10 ^-1^* |  | 1.2 | 0.4-4.6 | *7.0 x 10 ^-1^* | |
| ***LDHA*** | | | | | | | | | |  |
| Low^c^ | 28 |  | 1.0 |  | |  | 1.0 |  | | |
| *High* | 8 |  | 3.6 | 0.8-16.4 | *4.2 x 10^-2^* |  | 4.0 | 0.9-19.0 | ***5.0 x 10^-2^*** | |
| ***PGK1*** | | | | | | | | | |  |
| Low | 20 |  | 1.0 |  | |  | 1.0 |  | | |
| High | 16 |  | 9.9 | 1.2-83.5 | *3.5 x 10^-2^* |  | 8.6 | 1.4-24.3 | *2.5 x 10^-1^* | |
| ***ALDO*** | | | | | | | | | |  |
| Low | 30 |  | 1.0 |  | |  | 1.0 |  | | |
| High | 6 |  | 4.5 | 0.9-20.1 | *5.3 x 10^-2^* |  | 5.5 | 1.1-27.7 | *3.7 x 10^-1^* | |
| ***ENO1*** | | | | | | | | | |  |
| Low | 17 |  | 1.0 |  | |  | 1.0 |  | | |
| High | 19 |  | 3.0 | 0.6-15.6 | *1.9 x 10^-1^* |  | 4.2 | 0.6-27.6 | *1.3 x 10^-1^* | |
| ***GAPDH*** | | | | | | | | | |  |
| Low | 17 |  | 1.0 |  | |  | 1.0 |  | | |
| High | 19 |  | 2.6 | 0.5-13.4 | *2.5 x 10^-1^* |  | 2.3 | 0.4-13.0 | *4.0 x 10^-1^* | |
| ***SLC2A1*** | | | | | | | | | |  |
| Low | 30 |  | 1.0 |  | |  | 1.0 |  | | |
| High | 6 |  | 2.3 | 0.5-10.1 | *2.8 x 10^-1^* |  | 2.8 | 0.6-13.6 | *2.1 x 10^-1^* | |
| ***PKM*** | | | | | | | | | |  |
| Low | 11 |  | 1.0 |  | |  | 1.0 |  | | |
| High | 25 |  | 2.7 | 0.4-22.4 | *3.6 x 10^-1^* |  | 2.4 | 0.3-20.6 | *4.2 x 10^-1^* | |
| ***PFKP*** | | | | | | | | | |  |
| Low | 11 |  | 1.0 |  | |  | 1.0 |  | | |
| High | 25 |  | 0.7 | 0.1-3.9 | *7.1 x 10^-1^* |  | 0.6 | 0.1-3.6 | *6.1 x 10^-1^* | |
| ***HK2*** | | | | | | | | | |  |
| Low | 6 |  | 1.0 |  | |  | 1.0 |  | | |
| High | 30 |  | 2.6 | 0.1-10.1 | *4.7 x 10^-1^* |  | 0.6 | 0.0-1.6 | *9.8 x 10^-1^* | |

CI= confidence interval; HR= hazard ratio; FIGO stage= International Federation of Gynecology and Obstetrics stage.

a. Optimal cut off values were selected according to the ROC curve of the glycolysis FC score.

b. The analysis was performed considering the glycolysis FC score as continuous variable.

c. Optimal cutoff values were selected according to the ROC analysis in relation to the mean expression of genes obtained with the HG-Focus microarray.

d. Adjusted hazard ratio.

e. Cox proportional hazards model

f. These calculations were obtained in the multivariate analysis performed with *LDHA*. The values of FIGO obtained in the multivariate analysis with the other markers are not shown but are similar to these values.

g Univariate analysis was performed considering one variable for the analysis.

h. Multivariate analysis was performed considering gene expression and FIGO stage for the analysis.

**Supplementary Table 5**. Univariate and multivariate analyses for the disease-free survival of patients with CC with Cox proportional hazards models including the expression of glycolytic genes explored with HG-Focus microarray and FIGO clinical stage.

| **Covariates** |  |  | **Univariate analysis^g^** | | |  | **Multivariate analysis^h^** | | | |
| --- | --- | --- | --- | --- | --- | --- | --- | --- | --- | --- |
|  | **n** |  | **HR^d^** | **95% CI** | **p^e^** |  | **HR^b^** | **95% CI** | **p^e^** | |
| **FIGO** | | | | | | | | | |  |
| <IIA | 26 |  | 1.0 |  | |  | 1.0 |  | | |
| >IIB | 10 |  | 5.6 | 1.1-27.9 | *3.4 x 10^-2^* |  | 5.4^d^ | 1.1-26.3^d^ | *3.0 x 10^-2f^* | |
| **Glycolytic gene expression profile** | | | | | | | | | |  |
| FC < 1.95^a^ | 18 |  | 1.0 |  |  |  | 1.0 |  |  | |
| FC ≥ 1.95 | 18 |  | 2.4 | 0.6-9.0 | *2.5 x 10^-1^* |  | 2.5 | 0.7-11.2 | *2.0 x 10 ^-1^* | |
| Glycolysis FC score^b^ | 36 |  | 1.6 | 0.5-4.8 | *4.0 x 10^-1^* |  | 1.5 | 0.5-4.6 | *3.9 x 10 ^-1^* | |
| ***LDHA*** | | | | | | | | | |  |
| Low^c^ | 28 |  | 1.0 |  | |  | 1.0 |  | | |
| High | 8 |  | 3.6 | 0.9-13.7 | *5.0 x 10^-2^* |  | 4.2 | 1.1-16.6 | ***3.9 x 10^-2^*** | |
| ***PGK1*** | | | | | | | | | |  |
| Low | 20 |  | 1.0 |  | |  | 1.0 |  | | |
| High | 16 |  | 5.4 | 1.1-26.4 | *3.6 x10^-2^* |  | 9.9 | 1.5-24.3 | *1.6 x 10^-1^* | |
| ***ALDO*** | | | | | | | | | |  |
| Low | 30 |  | 1.0 |  | |  | 1.0 |  | | |
| High | 6 |  | 2.8 | 0.7-11.35 | *1.4 x 10^-1^* |  | 3.9 | 0.9-17.7 | *7.4 x 10^-1^* | |
| ***ENO1*** | | | | | | | | | |  |
| Low | 17 |  | 1.0 |  | |  | 1.0 |  | | |
| High | 19 |  | 3.6 | 0.7-17.8 | *1.2 x 10^-1^* |  | 3.6 | 0.7-17.8 | *1.2 x 10^-1^* | |
| ***GAPDH*** | | | | | | | | | |  |
| Low | 17 |  | 1.0 |  | |  | 1.0 |  | | |
| High | 19 |  | 2.0 | 0.5-8.0 | *3.2 x 10^-1^* |  | 1.5 | 0.3-6.6 | *5.7 x 10^-1^* | |
| ***SLC2A1*** | | | | | | | | | |  |
| Low | 30 |  | 1.0 |  | |  | 1.0 |  | | |
| High | 6 |  | 1.5 | 0.3-7.5 | *5.8 x 10^-1^* |  | 2.8 | 0.7-11.7 | *1.5 x 10^-1^* | |
| ***PKM*** | | | | | | | | | |  |
| Low | 11 |  | 1.0 |  | |  | 1.0 |  | | |
| High | 25 |  | 1.2 | 0.3-1.6 | *5.8 x 10^-1^* |  | 1.2 | 0.3-6.3 | *7.8 x 10^-1^* | |
| ***PFKP*** | | | | | | | | | |  |
| Low | 11 |  | 1.0 |  | |  | 1.0 |  | | |
| High | 25 |  | 0.6 | 0.3-7.5 | *5.3 x 10^-1^* |  | 0.6 | 0.1-2.3 | *3.7 x 10^-1^* | |
| ***HK2*** | | | | | | | | | |  |
| Low | 6 |  | 1.0 |  | |  | 1.0 |  | | |
| High | 30 |  | 1.5 | 0.8-11.75 | *7.1 x 10^-1^* |  | 1.3 | 0.1-10.4 | *8.1 x 10^-1^* | |

CI= Confidence interval; HR= hazard ratio; FIGO stage= International Federation of Gynecology and Obstetrics stage.

a. Optimal cut off values were selected according to the ROC curve of the glycolysis FC score.

b. The analysis was performed considering the glycolysis FC score as continuous variable.

c. Optimal cutoff values were selected according to the ROC analysis in relation to the mean expression of genes obtained with the HG-Focus microarray.

d. Adjusted hazard ratio.

e. Cox proportional hazards model.

f. These calculations were obtained in the multivariate analysis performed with *LDHA*. The values of FIGO obtained in the multivariate analysis with the other markers are not shown but are similar to these values.

g. Univariate analysis was performed considering one variable for the analysis.

h. Multivariate analysis was performed considering gene expression and FIGO stage for the analysis.

**Supplementary Table 6.** Expression of *LDHA* and *PFKP* by

qRT–PCR in clinical groups (n=58)

| **Clinical groups** | **Gene expression** | | |
| --- | --- | --- | --- |
|  | **n** | **Mean (ng)^a^ + SD** | ***p^b^*** |
| **Overall survival** | | | |
| ***LDHA*** | | | |
| Survival | 45 | 37.1 ± 30.2 | *9.0 x 10^-2^* |
| Dead | 13 | 54.9 ± 41.8 |  |
| ***PFKP*** | | | |
| Survival | 45 | 40.6 ± 33.6 | *3.5 x 10^-2^* |
| Dead | 13 | 66.6 ± 51.6 |  |
| **Disease-free survival (DFS)** | | | |
| ***LDHA*** | | | |
| DFS | 40 | 33.5 ± 25 | *9.0 x 10^-3^* |
| Non DFS | 18 | 58 ± 43.6 |  |
| ***PFKP*** | | | |
| DFS | 40 | 38.9 ± 33.9 | *2.8 x 10^-2^* |
| Non DFS | 18 | 63.2 ± 46 |  |

a. Mean absolute quantification with standard curve.

The expression was normalized to that of *RPS13.*

b. Mann–Whitney test.

SD=standard deviation.

**Supplementary Table 7.** Expression of LDHA and PFKP by IHC in cervical cancer.

| **Clinical groups** | **LDHA** | | | | | |  | **PFKP** | | | |
| --- | --- | --- | --- | --- | --- | --- | --- | --- | --- | --- | --- |
|  | **n** |  | **DOI ^a^** | **DE** | **FC^b^** | **p^c^** |  | **DOI ^a^** | **DE** | **FC^b^** | **p^c^** |
|  |  |  |  |  |  |  |  |  |  |  |  |
| HCT | 12 |  | 34,160 | 32,153 |  |  |  | 3,124 | 4,385 |  |  |
| CC | 18 |  | 147,387 | 108,499 | 4.3 | 1.2 x 10^-3^ |  | 84,935 | 63,105 | 27.2 | 7.6 x 10^-3^ |
| Metastatic | 6 |  | 356,409 | 146,719 | 10.4 | 3.2 x 10^-6^ |  | 133,660 | 82,216 | 42.7 | 1.7 x 10^-5^ |

a. DOI=mean integrated density.

b. Fold change (FC) was calculated with the median values as follows: clinical groups/HCT.

c. Mann–Whitney test.

CC= cervical cancer.

**Supplementary Table 8**. Hazard ratio analyses of patients with CC with Cox proportional hazards models including the expression of glycolytic genes and FIGO clinical stage by qRT–PCR.

| **Covariates^a^** | |  | |  | | **Univariate analysis^e^** | | | | | | | |  | **Multivariate analysis^f^** | | | | | |
| --- | --- | --- | --- | --- | --- | --- | --- | --- | --- | --- | --- | --- | --- | --- | --- | --- | --- | --- | --- | --- |
|  |  | **n** | |  | | **HR^c^** | | **95% CI** | | ***p*^d^** | | | |  | **HR^c^** | **95% CI** | ***p*^d^** | | | |
| **Overall Survival** | | | | | | | | | | | | | | | | | | | |  |
| *LDHA* | | | | | | | | | | | | | | | | | | | |  |
| FIGO stage <IIA^a^ | | 29 | |  | | 1.0 | |  | | | | | |  | 1 |  | | | | |
| FIGO stage >IIB | | 29 | |  | | 2.6 | | 0.9-8.1 | | 8.7 x 10^-2^ | | | |  | 1.7 | 0.5-5.5 | 1.2 x 10^-1^ | | | |
| Low^b^ | | 23 | |  | | 1.0 | |  | | | | | |  | 1 |  | | | | |
| High | | 35 | |  | | 5.6 | | 1.2-26.3 | | 2.9 x 10^-2^ | | | |  | 5.0 | 1.0-23.8 | 4.4 x 10^-2^ | | | |
| *PFKP* | | | | | | | | | | | | | | | | | | | |  |
| FIGO stage <IIA | | 29 | |  | | 1 | |  | | | | | |  | 1 |  | | | | |
| FIGO stage >IIB | | 29 | |  | | 2.6 | | 0.9-8.1 | | | | 8.7 x 10^-2^ | |  | 2.0 | 0.6-6.7 | | | 4.4 x 10^-2^ | |
| Low | | 26 | |  | | 1.0 | |  | | | | | |  | 1 |  | | | | |
| High | | 32 | |  | | 5.4 | | 1.2-24.6 | | 2.8 x 10^-2^ | | | |  | 5.0 | 1.1-22.9 | 3.7 x 10^-2^ | | | |
| *LDHA/PFKP* ^g^ | | | | | | | | | | | | | | | | | | | |  |
| FIGO stage <IIA | | 29 | |  | | 1 | |  | | | | | |  | 1 |  | | | | |
| FIGO stage >IIB | | 29 | |  | | 2.6 | | 0.9-8.1 | | | | 8.7 x 10^-2^ | |  | 2.3 | 0.8-6.6 | | | 1.3 x 10^-1^ | |
| Low/one high | | 34 | |  | | 1 | |  | | | | | |  | 1 |  | | | | |
| Two high | | 24 | |  | | 6.7 | | 1.7-24.4 | | 7.0 x 10^-3^ | | | |  | 6.4 | 1.7-26.6 | 7.0 x 10^-3^ | | | |
| **Disease-free survival** | | | | | | | | | | | | | | | | | | |  |  |
| *LDHA* | | | | | | | | | | | | | | | | | | |  |  |
| FIGO stage <IIA | | 29 | |  | | 1 | |  | | | | |  | 1 |  | | | |  |  |
| FIGO stage >IIB | | 29 | |  | | 2.6 | | 0.9-7.6 | | 7.3 x 10^-2^ | | |  | 2.5 | 0.9-7.0 | 8.3 x 10^-2^ | | |  |  |
| Low | | 23 | |  | | 1 | |  | | | | |  | 1 |  | | | |  |  |
| High | | 35 | |  | | 5.5 | | 1.5-19.7 | | 9.0 x 10^-3^ | | |  | 4.8 | 1.3-17.3 | 1.8 x 10^-2^ | | |  |  |
| *PFKP* | | | | | | | | | | | | | | | | | | |  |  |
| FIGO stage <IIA | | 29 | |  | | 1 | |  | | | | |  | 1 |  | | | |  |  |
| FIGO stage >IIB | | 29 | |  | | 2.6 | | 0.9-7.6 | | | | 7.3 x 10^-2^ |  | 2.1 | 0.8-6.0 | | 1.5 x 10^-2^ | |  |  |
| Low | | 26 | |  | | 1 | |  | | | | |  | 1 |  | | | |  |  |
| High | | 32 | |  | | 5.0 | | 1.4-17.2 | | 1.8 x 10^-2^ | | |  | 4.5 | 1.3-15.8 | 1.7 x 10^-2^ | | |  |  |
| *LDHA/PFKP* ^g^ | | | | | | | | | | | | | | | | | | |  |  |
| FIGO stage <IIA | | 29 | |  | | 1 | |  | | | | |  | 1 |  | | | |  |  |
| FIGO stage >IIB | | 29 | |  | | 2.6 | | 0.9-7.6 | | 7.3 x 10^-2^ | | |  | 2.6 | 0.9-7.6 | 7.3 x 10^-2^ | | |  |  |
| Low/one high | | 34 | |  | | 1 | |  | | | | |  | 1 |  | | | |  |  |
| Two high | | 24 | |  | | 7.7 | | 2.2-23.5 | | 1.0 x 10^-3^ | | |  | 7.1 | 2.2-27.1 | 1.0 x 10^-3^ | | |  |  |

a. FIGO stage analysis.

b. Optimal cut off values were selected according to the ROC analysis in relation to the expression of *LDHA* and *PFKP*

obtained with qRT–PCR.

c. Adjusted hazard ratio.

d. Cox proportional hazards model.

e. Univariate analysis was performed considering one variable for the analysis.

f. Multivariate analysis was performed considering gene expression and FIGO stage for the analysis.

g. Low/one high= downregulation of two genes or upregulation of one gene; Two high= upregulation of *LDHA* and *PFKP.*

CI= confidence interval; HR = hazard ratio; FIGO stage= International Federation of Gynecology and Obstetrics stage.

**Supplementary Table 9**. Hazard risk analyses of patients with CC included in the TCGA database using Cox proportional hazards models including the expression of *LDHA* and *PFKP* genes and FIGO clinical stage.

| **Covariates^a^** |  |  | **Univariate analysis^e^** | | | |  |  | **Multivariate analysis^f^** | | | | |
| --- | --- | --- | --- | --- | --- | --- | --- | --- | --- | --- | --- | --- | --- |
|  | **n** |  | **HR^c^** | **95% CI** | ***p*^d^** | |  |  | **HR^c^** | **95% CI** | ***p*^d^** | | |
| **Overall Survival** | | | | | | | | | | | | |  |
| *LDHA* | | | | | | | | | | | | |  |
| FIGO stage I-II^a^ | 230 |  | 1.0 |  | | |  |  | 1 |  | | | |
| FIGO stage III-IV | 65 |  | 2.4 | 1.4-3.9 | 1.0 x 10^-3^ | |  |  | 2.1 | 1.3-3.4 | 4.0 x 10^-3^ | | |
| Low^b^ | 177 |  | 1.0 |  | | |  |  | 1 |  | | | |
| High | 118 |  | 2.1 | 1.3-3.3 | 2.0 x 10^-3^ | |  |  | 1.9 | 1.2-3.0 | 1.1 x 10^-2^ | | |
| *PFKP* | | | | | | | | | | | | |  |
| FIGO stage <IIA | 230 |  | 1 |  | | |  |  | 1 |  | | | |
| FIGO stage >IIB | 65 |  | 2.4 | 1.4-3.9 | | 1.0 x 10^-3^ |  |  | 2.2 | 1.3-3.6 | | 2.0 x 10^-3^ | |
| Low | 169 |  | 1.0 |  | | |  |  | 1 |  | | | |
| High | 126 |  | 2.2 | 1.4-3.6 | 1.0 x 10^-3^ | |  |  | 2.0 | 1.3-3.4 | 2.0 x 10^-3^ | | |
| *LDHA/PFKP* ^g^ | | | | | | | | | | | | |  |
| FIGO stage I-II | 230 |  | 1 |  | | |  |  | 1 |  | | | |
| FIGO stage III-IV | 65 |  | 2.4 | 1.4-3.9 | | 1.0 x 10^-3^ |  |  | 2.0 | 1.2-3.3 | | 7.0 x 10^-3^ | |
| Low | 117 |  | 1 |  | | |  |  | 1 |  | | | |
| One high | 113 |  | 2.3 | 1.3-4.2 | | 7.0 x 10^-3^ |  |  | 2.1 | 1.2-3.9 | | 1.5 x 10^-2^ | |
| Two high | 65 |  | 3.7 | 2.0-7.0 | 5.6 x 10^-5^ | |  |  | 3.2 | 1.7-6.1 | 3.4 x 10^-4^ | | |

a. FIGO stage analysis.

b. Optimal cut off values were selected according to the ROC analysis in relation to the expression of *LDHA* and *PFKP*

obtained of TCGA database.

c. Adjusted hazard ratio.

d. Cox proportional hazards model.

e. Univariate analysis was performed considering one variable for the analysis.

f. Multivariate analysis was performed considering gene expression and FIGO stage for the analysis.

g. Low/one high= downregulation of two genes or upregulation of one gene; Two high= upregulation of *LDHA* and *PFKP.*

CI= confidence interval; HR = hazard ratio; FIGO stage= International Federation of Gynecology and Obstetrics stage.
